# Rapid Evaluation of CRISPR Guides and Donors for Engineering Mice

**DOI:** 10.3390/genes11060628

**Published:** 2020-06-08

**Authors:** Elena McBeath, Jan Parker-Thornburg, Yuka Fujii, Neeraj Aryal, Chad Smith, Marie-Claude Hofmann, Jun-ichi Abe, Keigi Fujiwara

**Affiliations:** 1 Department of Endocrine Neoplasia and Hormonal Disorders, University of Texas MD Anderson Cancer Center, Houston, TX 77030, USA; MHofmann@mdanderson.org; 2 Department of Genetics, University of Texas MD Anderson Cancer Center, Houston, TX 77030, USA; neerajkaryal@gmail.com (N.A.); chadsmit@mdanderson.org (C.S.); 3 Department of Cardiology, University of Texas MD Anderson Cancer Center, Houston, TX 77030, USA; yfujii@houstonmethodist.org (Y.F.); JAbe@mdanderson.org (J.-i.A.); KFujiwara1@mdanderson.org (K.F.)

**Keywords:** CRISPR/Cas9, gene targeting, genetic engineering, blastocyst, silent mutation

## Abstract

Although the Clustered Regularly Interspaced Short Palindromic Repeats (CRISPR)/ CRISPR associated protein 9 (Cas9) technique has dramatically lowered the cost and increased the speed of generating genetically engineered mice, success depends on using guide RNAs and donor DNAs which direct efficient knock-out (KO) or knock-in (KI). By Sanger sequencing DNA from blastocysts previously injected with the same CRISPR components intended to produce the engineered mice, one can test the effectiveness of different guide RNAs and donor DNAs. We describe in detail here a simple, rapid (three days), inexpensive protocol, for amplifying DNA from blastocysts to determine the results of CRISPR point mutation KIs. Using it, we show that (1) the rate of KI seen in blastocysts is similar to that seen in mice for a given guide RNA/donor DNA pair, (2) a donor complementary to the variable portion of a guide integrated in a more all-or-none fashion, (3) donor DNAs can be used simultaneously to integrate two different mutations into the same locus, and (4) by placing silent mutations about every 6 to 10 bp between the Cas9 cut site and the desired mutation(s), the desired mutation(s) can be incorporated into genomic DNA over 30 bp away from the cut at the same high efficiency as close to the cut.

## 1. Introduction

The CRISPR/Cas technique has rapidly become widespread in use due to its ability to produce targeted and specific mutations in the genome with relative ease. Since gene editing in organisms such as mice and other mammals is usually much more expensive and time consuming than it is for cell lines or smaller, shorter lived organisms, the financial advantages of using CRISPR systems have driven the widespread adoption of the procedure by nearly all transgenic facilities. In turn, determining the effectiveness of the different methods and components used for CRISPR has become a concern. Major factors that affect the outcome of a CRISPR project are the efficiency and form of guide RNA used to direct cutting, the repair pathway, proper donor DNA incorporation and cell viability.

A common problem in applying the CRISPR system stems from the lack of efficiency with which a guide RNA may direct Cas9 to cut or, if it works well, with which a donor DNA sequence incorporates into the genome. In addition, the preparations of the guides, donors and other components used for CRISPR can often be chemically compromised, which would lead to lethality upon injection into the mouse embryo [1].

Several online programs are available that predict the efficiency with which different guides direct cutting. These are quite useful in estimating how well a chosen guide will work, but in practice, actual guide efficiency appears to vary by as much as 20% or more from its predicted efficiency [2]. One may test the effectiveness of a guide using purified CRISPR components in a test tube [3,4]. This approach can be useful for eliminating those that are ineffective. However, in our hands, the way the guide RNA is made, stored and used, as well as the source and condition of Cas9 can appreciably alter the apparent ability of a guide to direct the cut. One could also use a cell line to test a guide. However, it has been shown that a guide which works effectively in one cell type may not work well in another, presumably due to various factors such as masking of genomic DNA by nucleosomes that prevents good binding by Cas9 [5]. So, despite its efficiency in a cell line, a guide may not direct cutting efficiently in the zygote.

None of the above approaches can be used to predict how efficiently a donor DNA will incorporate its sequence into the genome. A donor DNA’s characteristics, such as single versus double strand [6], length [7], orientation relative to the sgRNA [8], and the distance of the desired mutation from the cut site [9,10], as well as the state of the cell [11] and target genomic site [12], play a large role in the ability of a mutation to integrate. Thus, determining how well a sgRNA directs cutting is not sufficient to predict success in integrating that mutation in vivo.

To address these issues, many have used blastocysts developed from CRISPR treated zygotes to determine the ability of their guides to direct cutting [13,14,15] and donor DNA to integrate [14,15,16], before spending the time and money injecting these components to make genomically engineered mice. However, no extensively detailed and simple CRISPR blastocyst protocol, with results shown to be replicated in mice has yet been published and we felt there was a need for such a guide. Here we have devised and described, step-by-step, a simple in vivo test of the sgRNA, donor DNA and other injection components that can be done prior to injecting a large number of embryos for implantation. By allowing CRISPR components-injected zygotes to develop to the blastocyst stage, sequencing their DNA, and using Synthego’s Inference of CRISPR Edits (ICE) program, we were able to predict within a few days what will happen to the genomic DNA in mice developed from zygotes injected with the same CRISPR components and under the same conditions. If the zygotes fail to develop normally, the results of injecting each component separately can then pinpoint which component is causing the problem.

Using our optimized blastocyst technique, we found that integration of donor containing sequence complementary to the guide appeared to occur in a more all-or-none fashion than when using donor containing the same sequence as the guide, and that it was possible to get blastocysts containing two mutations in the same locus after one injection with two donors. Normally, the efficiency with which a donor integrates a point mutation drops, at 30 bases away from the cut, to about 10% of the value found for integration of the same mutation at the cut [9,10]. However here, we were able to show that silent mutations about every 10 bp or less between the cut and desired mutation allowed for similar high integration efficiencies of that mutation, regardless of being from a few base pairs to over 30 bp from the cut.

## 2. Materials and Methods

### 2.1. Blastocysts Assay Overview 

In the process of generating CRISPR engineered mice for several different ongoing projects related to inflammatory cardiovascular diseases, we attempted to improve the outcome by developing and testing our blastocyst method prior to making the mice. Four projects intended to produce a point mutation, each in a different gene, were chosen: *Magi1* [17,18], *Mapk7* (Erk5) [19], *Atox1* [20], and *Terf2ip* [21], all of which play various roles in the pathogenesis of atherosclerosis. Three mouse genes, *Magi1*, *Mapk7* and *Atox1*, were used to test the blastocyst method with a single donor DNA for each guide RNA. Zygotes were also injected with dual donors simultaneously to see if both donor mutations altering the same serine in the *Terf2ip* mouse gene could be incorporated, each into different copies of the same chromosome, to make blastocysts and mice with both mutations at once. All animal experiments described in this paper and mouse maintenance were according to the guidelines set out by the NIH Guide for the Care and Use of Laboratory Animals and approved by the Institutional Animal Care and Use Committee of University of Texas MD Anderson Cancer Center (Registration numbers 00001652—approval in 2016 and 2019, 00001109—approval in 2017 and 2020, and 00000921—approval in 2016 and 2019) and Texas A&M Institute of Biomedical Technologies (Registration numbers 2014-0231, 2017-0154; approved in 2014 and 2017 respectively).

Guide RNAs and donor DNAs were designed and used to alter a single amino acid for each gene (see “Design and preparation of sgRNA” below for details). These were serine 733 to alanine for Magi1, serine 496 to alanine for Erk5 (*Mapk7* gene), lysine 3 to arginine for Atox1, and serine 202 to either alanine or aspartic acid for Terf2ip. For *Magi1*, three guide RNAs were generated to direct cutting near the desired amino acid mutation site and two guides each for both *Mapk7* and *Atox1*. One guide was used for *Terf2ip*.

One single stranded donor, containing the same sequence (target [22]) as the guide’s variable region (unique “spacer” [22]), was designed for each guide except in the case of guide RNAs gR8179 for *Magi1* and gR2077 for *Terf2ip*. For *Magi1* gR8179, both a donor containing the same sequence (M79) and a donor containing the complementary sequence (M79R) to the guide’s variable region were designed. For *Terf2ip* gR2077, both a donor coding for alanine (A) and a donor coding for aspartic acid (D) were designed. The sequence of the guides and donors used are listed in Table S1. 

### 2.2. Design and Preparation of Single Guide RNA

To design guides with both reduced off-target effects and the best chance of efficient cutting, we started with a 110 bp genomic sequence centered on the codon encoding the amino acid to be altered. To select guides calculated to have fewer off-target effects, the 110 bp sequence was run through MIT’s CRISPR design program for mouse (http://crispr.mit.edu/), subsequently replaced with CRISPOR (http://crispor.tefor.net/crispor.py [23]), and only those sequences with a score above 50 were considered further. It should be noted that working with mice and other sexual organisms provides the advantage over cell lines that most off-targets can be bred out. 

To increase the chance of selecting guides with a better ability to direct the cut, the 110 bp genomic sequence was also run through the Sequence Scan for CRISPR program (http://crispr.dfci.harvard.edu/SSC/ [2]). The Sequence Scan for CRISPR cutting efficiency scores were converted to percentages by plugging them into the formula for the best fit line (y = 29.06x + 56.239 R^2^ = 0.786) for data from the Supplemental Table_4.xlsx in the article by Xu et al. [2]. Data from Figure 5 in the article by Elliott et al. [10] was used to construct a best fit bell-shaped curve for percent successful homology directed repair (HDR) versus distance from cut. A combined average of the SSC score percent and the percent HDR efficiency was calculated for each guide with an off-target score above 50 and those with the highest values were selected. 

Since it is possible that strong secondary structure between the 19–20 base variable region of the guide with itself or with the non-varying region might interfere with the guide binding normally to the genomic DNA or Cas9 respectively, we tried to avoid guides with a variable spacer region having secondary structure with a melting temperature (Tm) well above 37 °C within itself or with the constant scaffold region of the guide. Finally, to facilitate RNA production of the guides using the T7 promoter, those guides with a G in the first or second position from the 5′ end were chosen. If the first 5′ base was a G, the full 20 base variable region was used. If G was not in the first position but was in the second position, the first 5′ base, was removed to make a 19 base variable region containing guide. For each gene, the 3 sequences best fitting these criteria and having the highest averaged efficiency of predicted cutting with successful HDR were selected for guide production. 

Each of the best three 20 or 19 base sequences was then inserted into the N region in the following T7 cassette sequence, gaaatTAATACGACTCACTATAGNNNNNNNNNNNNNNNNNNNGTTTTAGAGCTAGAAATAGCAAGTTAAAATAAGGCTAGTCCGTTATCAACTTGAAAAAGTGGCACCGAGTCGGTGCt_X_. The gaaatTAATACGACTCACTATA sequence is the T7 promoter sequence, GNNNNNNNNNNNNNNNNNNN represents the previously selected 20 or 19 base variable recognition sequence for the genomic DNA including its 5′ G, GTTTTAGAGCTAGAAATAGCAAGTTAAAATAAGGCTAGTCCGTTATCAACTTGAAAAAGTGGCACCGAGTCGGTGC is the sequence for the invariant, “scaffold” [22] portion of the sgRNA with U replacing T, and the multiple Ts (t_X_) that follow it encode the T7 termination sequence. The T7 termination sequence should not be necessary in this case as the T7 polymerase will run off the end. Enough 3′ terminal Ts were typically added to produce a T7 cassette sequence of around 120 bases maximum. A sequence ending with just one T (transcribed as U in the RNA) worked to cut genomic DNA with equal efficiency as those with 3 or 4 terminal Ts.

SnapGene (http://www.snapgene.com/) was the primary program used for analysis of DNA Sanger sequences and GeneRunner (Version 5.0.46 Beta, http://www.generunner.net/) was used for initial Tms and secondary structure prediction.

The forward and reverse ssDNA sequences of these T7 cassettes were ordered as ~120 base oligonucleotides (oligos) from Sigma Aldrich (Saint Louis, MO, USA), either dry or resuspended in Sigma’s TE buffer at 100 μM. If ordered dry, the oligo was then resuspended at 100 μM in 10 mM Tris, pH 7.4, 0.1 mM EDTA, in RNAse, DNAse free, sterile Molecular Grade water. To anneal the two strands, 10 μL of forward strand and 10 μL of reverse strand were mixed (1:1), heated to 95 °C for 5 min, slowly cooled to below 37 °C over the course of one hour and stored at −20 °C until used for transcription. 

sgRNAs were originally produced using standard in vitro transcription. The MEGA Shortscript AM1354 kit (ThermoFisher/Invitrogen, Waltham, MA, USA) was used to generate sgRNA, which was then purified using the MEGAclear kit AM1908 (ThermoFisher/Invitrogen). After RNA recovery, the purity of the RNA produced was assessed using the RNA 6000 Pico kit applied to the Agilent Technologies Bioanalyzer 2100.

Chemically synthesized sgRNAs were obtained from Synthego (Synthego, Redwood City, CA, USA, http://www.synthego.com/). Synthego sgRNAs no longer restrict the guides to having a 5′ G in the variable sequence, are much smoother to inject and appears to be less toxic based on subsequent birth rates observed. Overall, rates of cutting and point mutation integration when using Synthego guides appear to be increased over in-house produced sgRNA.

### 2.3. Design and Preparation of Single Strand Donor DNA

Donor DNAs were initially designed to be 110 bases long, single stand, and centered on the mutation(s) giving rise to the desired amino acid change. The strand chosen contained the same sequence as the 20 or 19 base variable region of the guide RNA, with T replacing U. In one case, a donor was made using the strand containing the complement to the variable region. Based on the results of a recent study [24], we shortened the donor for the Terf2ip experiment to contain 37 bases of homology beyond the cut on one side, and 37 bases of homology beyond the desired mutation on the other side, which led, when including the region between, to a donor with a total length of 81 bases.

Using GenScript’s mouse codon frequency tables (GenScript Codon Usage Frequency Table Tool, https://www.genscript.com/tools/codon-frequency-table), the codon used for the desired amino acid change was selected to have an about equal or slightly higher frequency tRNA than the original codon, if possible, to prevent more frequent premature translation termination by the ribosome. If this was not feasible, several codons were silently changed to make that run of codons have a similar overall frequency as the original set of codons. Codons increasing the frequency of local hairpins with a Tm above 37 °C were also avoided, when possible.

**Figure 1:**
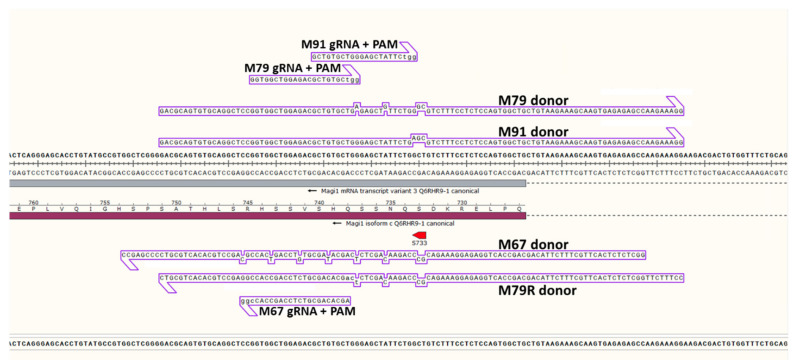
Magi1 guide and donor pair sequences. Four guide/donor pairs were used to alter serine 733 in the mouse *Magi1* gene to alanine. The M67, M79 and M91 guides had donors containing the variable region of the guide sequence (except guides had U in place of T) and these guide RNA regions are shown with the adjacent PAM in lower case. We also used the M79 guide with another donor, M79R donor, containing sequence complementary to the variable region of the M79 guide. Donor point mutations are shown out of line with the main donor sequence.

To prevent or reduce recutting upon donor sequence incorporation [9,25], one of the GG bases of the donor’s PAM was replaced with a silent mutation if possible, and if not, one of the 4 bases 5′ of the PAM was silently altered (Figure 1). In order to increase the efficiency of donor sequence incorporation when the cut site was far from the desired amino acid altering point mutation(s), silent mutations were placed about every 10 bases in the sequence between the cut site and the amino acid mutation site. As with the amino acid altering mutations, the replacement base for each silent point mutation was chosen to produce a codon with a tRNA usage frequency about equal to or slightly exceeding the unaltered codon, as well as to prevent an increase in the frequency of local hairpins with a Tm above 37 °C.

To help prevent the guide RNA from binding the target region in the donor DNA and forming a duplex that can possibly be cut by the guide RNA/Cas9 complex [25], the strand that had the same sequence as the guide variable spacer region, with T replacing U, was usually chosen. However, for the M79 guide, we also used the donor strand containing sequence complementary to the guide’s variable spacer region. In all cases in which Cas9 protein was used, we first generated the sgRNA/Cas9 RNP complex by adding the guide RNA to Cas9 and incubating at room temperature for 10 minutes, prior to adding donor DNA to the mixture. Doing so reduced binding of the guide RNA to the complementary donor DNA.

Once the donor sequence had been designed, donors were ordered as Ultramers from Integrated DNA Technologies using the Lab-Ready Oligo Service (resuspended at 100 µM concentration in IDTE (1X TE buffer), pH 8.0, https://www.idtdna.com/pages/products/dna-rna/custom-preparative-services).

### 2.4. Zygote Preparation, Injection, Culture and Lysis

Twenty-five to twenty-eight day-old female C57BL/6N mice (Charles River Laboratories, Wilmington, MA, USA) were superovulated using standard procedures [26] and then mated 1:1 to C57BL/6N male mice (3–8 months of age). Pronuclear stage embryos were isolated from mated females and injected with the various CRISPR mixtures using standardized pronuclear injection procedures [1]. All injection mixtures included sgRNA, one or two single strand oligo donor DNAs paired to the sgRNA (Table S1), and either Cas9 mRNA or protein (Table S2). Each guide RNA/Cas9 complex with its donor DNA was injected into 20–30 single-cell embryos to produce 8–10 blastocysts 3 to 4 days later. We saw no obvious difference in our results between experiments with Cas9 mRNA and protein, or the different component concentrations in the range of concentrations we used. However, the general consensus is that Cas9 protein is less likely to generate mosaicism than mRNA, so we elected to continue using protein.

After injection, embryos were cultured in pH equilibrated KSOM +AA (Millipore-Sigma) in a humidified incubator (Cook, Inc., Bloomington, IN, USA) with 5% CO_2_, 5% O_2_ and 90% N_2_ for 3 to 3.5 days until reaching the blastocyst stage. At that time, each embryo was individually placed into a 0.2 mL PCR tube (Applied Biosystems, Foster City, CA, USA) containing 2.5 μL Extraction Reagent lysis solution from the Extracta DNA Prep kit (Quanta Biosciences, Beverly, MA, USA), covered with ~10-20 µL of embryo-tested mineral oil (Millipore-Sigma, St. Louis, MO, USA), and either used immediately or stored at −80 °C for later use. The blastocyst lysate was heated at 95 °C for 30 min in a PCR cycler and cooled to room temperature. 2.5 μL of Stabilization Buffer (Extracta DNA Prep kit, Quanta Biosciences) were added to the blastocyst lysate and mixed by inversion, flicking and centrifuging briefly with a microcentrifuge. This mixing/centrifugation step was repeated once. Stabilized blastocyst lysates were either used immediately or stored at −20 °C.

### 2.5. Primers

Typically, two sets of nested primers were designed for each point mutation (Table S3). Primer sequences were chosen which had a Tm of around 65 °C to 66 °C (range of 62 °C to 69 °C) using NEB’s Tm calculator for Q5 (https://tmcalculator.neb.com/#!/main), kept primer partners within 5 °C of each other, had no runs of 3 or more of the same base, particularly G or C close to the 3′ end, had between 40% to 60% GC content, and started at the 3′ end with usually C, otherwise G or A. Primers were checked using Gene Runner (http://www.generunner.net/) to make sure they had no strong secondary structure, particularly those leading to primer dimers, extending hairpins, and amplification at sites close to the desired genomic region being amplified. Primers were designed such that the final inner primer set generated an oligonucleotide of about 200 to 400 bp in length with the mutation located about half way through. This was done to obtain a high-quality Sanger sequencing read around the mutation site. The inner most primer on the side closer to the desired mutation rather than the primer closer to the cut site was then also used to sequence the oligo. We chose to use a primer upstream of the point mutations rather than upstream of where indels may first occur so that the height of the single or double base containing peaks in the Sanger chromatogram at each point mutation location could be clearly detected. Otherwise, the overlapping peaks caused by an indel induced shift would obscure the point mutation peaks.

### 2.6. PCR

PCR was performed using Q5 polymerase following NEB’s directions for 25 μL final product, for each blastocyst. When 8 or fewer blastocysts were to be assessed, the separate Q5 components were mixed, while for larger numbers of blastocysts, we used Q5 2X MasterMix. One μL of the Extracta-lysed-and-stabilized blastocyst lysate, or first PCR product to be used for the second nested PCR, was added as template. Primer annealing temperatures were calculated using NEB’s Tm calculator for Q5 and an extension time based on 30 s per kilobase (kb) was used, as recommended by NEB for complex DNA. We found that 60 cycles for each PCR worked for the majority of blastocyst samples. Recently we observed that an elongation time of 1 min per kb for 60 cycles or 2 to 3 min per kb for 30 cycles increased the chance of getting a strong, sequenceable band, as long as the fragment being amplified was short (<500 bp).

PCR products were run on a 2% agarose gel containing GelGreen (Biotium, Fremont, CA, USA, cat. no. 41005) diluted to 1X, and bands visualized by UV on a FluorChem 8900 imager (Alpha Innotech, San Leandro, CA, USA). If a band at or near the appropriate size was seen, 7.5 μL of that PCR product was added to 3 μL of ExoSAP-IT (Applied Biosystems, cat. no. 75001.200.UL), heated for 5 min at 37 °C, and 1 min at 80 °C to clean up the DNA in preparation for sequencing. The resulting 10.5 μL of sample along with the appropriate primer were sent off for sequencing to Eurofins Genomics (Louisville, KY, USA).

### 2.7. Analysis

#### ICE

The percent of wildtype (WT), indels and point mutation sequence were calculated using Synthego’s ICE programs (https://ice.synthego.com/#/). The ICE KO program, which calculates the amount of each type of indel, takes into account the presence of point mutations. Further, the ICE KI program does not require a homozygous Sanger sequence of the desired point mutations. The ICE program point mutation calculations are not as accurate as the indel calculations since only one or a few peaks can be analyzed. Although both these issues may lead to a less precise quantification, the ICE KI results were sufficient to predict outcomes in mice from those of the blastocysts.

It has been recommended to use an inherent Sanger sequencing error rate for good reads of ≤7.25% in detecting single point mutations [27]. Given that our sequences also usually included indels, we chose a maximum error rate of 8% when totaling point mutations, WT and indels percentages together.

Because the ICE point mutation program is not intended to quantify two donors at once, when analyzing the dual donor injected blastocysts, we ran the Sanger sequence ab1 files through the program twice, once with one donor DNA sequence and once with the alternate donor sequence. We manually inspected the peak heights of the point mutations compared to WT in each Sanger chromatogram to determine whether only one donor sequence appeared to have been integrated or whether the peak was composed of a mixture of the two. If only one appeared to be integrated, we selected the values from the ICE KI program in which that donor sequence was used. When both donor sequences were integrated in a single blastocyst, we calculated the average of the values obtained separately from each donor’s integrated sequence in the KI program.

The comparison of integration efficiency was done one of two ways. Bar graphs were made for each set of blastocysts treated with the same guide and donor pair where each bar shows the quantified percent WT, indels and desired point mutations for a single blastocyst. Bars were placed in order from those blastocysts containing the least percent of altered genomic DNA to those with the most. Alternatively, bar graphs of the ratio of desired KI mutation to total cut (KI mutation/KI mutation + indel) for each blastocyst were made, again, creating one graph for each set of blastocysts treated with the same guide and donor pair. Results for each blastocyst were ordered from those with the lowest ratio of desired KI mutation/total cut to those with the highest.

### 2.8. Mice

After reviewing the results of the blastocyst test, a guide and donor pair with high efficiency for point mutation integration was chosen and used to generate mouse lines. Pronuclear stage C57BL/6N embryos were isolated from superovulated and mated females and injected with the verified mixture of CRISPR components using standardized pronuclear injection procedures [1]. Surviving embryos were implanted as one-cell embryos on either the day of injection or as 2-cell embryos the following day into day 0.5 pseudopregnant CD-1 females (Charles River Laboratories) for gestation [28]. All surviving pups were genotyped after PCR by gel electrophoresis and sequencing to determine the amount of integration of the correct mutations and lack of any additional unwanted mutations surrounding the site of homology directed repair (HDR). For each gene, we successfully generated mice containing the desired amino acid mutation.

## 3. Results

### 3.1. General Strategy

For several CRISPR projects in which a single amino acid mutation was engineered into mice, we chose to test the CRISPR components using a quick method to inject and then produce mutated blastocysts which would then be analyzed to predict the efficiency of those components in generating the mice. Three projects were chosen, each altering a single amino acid in the gene of either *Magi1*, *Mapk7*, or *Atox1*. Data from each were used to optimize the steps of the blastocyst DNA amplification and sequencing procedure and to test the reproducibility of the results. Either 2 or 3 different single guide RNA (sgRNA) + single strand DNA (ssDNA) donor pairs were used for each gene, and the integration efficiencies of each pair compared. For one of the *Magi1* guides, we also compared integration efficiencies of a ssDNA donor containing the sequence matching the sgRNA to that of a ssDNA donor containing the sequence complementary to the sgRNA.

Once established, the procedure was additionally used to determine if two donor DNAs injected simultaneously could mutate the same amino acid into two different amino acids in the genome of the same blastocyst or several blastocysts in one experiment, before proceeding to use the same approach on mice.

The following is a detailed, step-by-step description of the optimized protocol.

### 3.2. Method Outline

**Figure 2:**
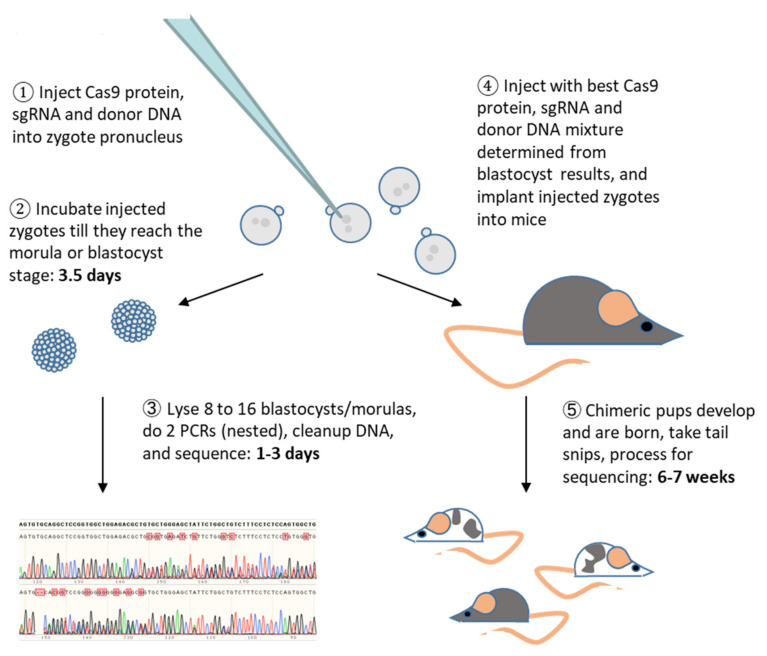
Workflow. Zygotes injected with CRISPR components are developed first to the blastocyst stage and used to determine the efficiency of the components. If results from the blastocysts are good, new zygotes are injected with the same CRISPR mixture and used to make mice.

The general procedure we used (Figure 2), with key points, is as follows:

1. Design and make or buy guide RNA and donor DNA.
2. Design and make or buy guide RNA and donor DNA.
3. Design a minimum of 2 sets of optimal primers to perform PCR around the CRISPR cut site and insertion site, one set inside of the other, for eventual use in nested PCR. Center the oligo made by the inner primers on the desired point mutations and include the guide cut region. Each primer for the inner set should be roughly 100 to 200 base pairs away from the closest point mutation or cut point, to give a final product of about 200 to 400 base pairs. This will produce the cleanest, strong, clear Sanger sequence peaks in the region containing the desired mutations to be analyzed. We typically chose outer primers to give a fragment of about 400 to 700 base pairs in size, to save on PCR time.
4. Inject Cas9, guide RNA and donor DNA into mouse single cell embryos and incubate for 3 to 3.5 days until they reach the blastocyst stage. We usually injected enough embryos to produce at least 8 blastocysts for each guide/donor pair, though not all blastocyst preparations give sequenceable PCR product. Morulae frequently also give usable results. With a good guide and donor pair producing above 50% donor sequence integration on average, as few as 4 embryos can be used, though we suggest using 8 or more.
5. Pick up blastocysts in the smallest volume of culture medium possible and lyse in a maximum of 10 μL final volume of lysing solution under ~10–20 μL mineral oil (to reduce evaporation). We typically used 2.5 μL of Extraction Reagent lysis solution (Quanta Biosciences). The solution of lysed blastocysts was mixed well by flicking and briefly centrifuging (microcentrifuge, repeated once), heated at 95 °C for 30 min, and then neutralized with an equivalent amount of Stabilization Buffer per manufacturer’s instructions. Homemade lysing solution gave similar results.
6. Perform PCR with the outer set of primers, using a high-fidelity polymerase as recommended by the manufacturer, except increase elongation time by either 4 times the recommended, or by 2 times and use 60 cycles instead of the usual 30–35 cycles. We ran a 25 μL reaction using Q5 polymerase (New England Biolabs, Ipswich, MA) and 1 μL of the lysed blast mixture. To get consistent results, it is important to mix the lysed blastocyst solution well but gently and spin it down before adding it to the PCR mixture.
7. Repeat the PCR with the inner set of primers and 1 μL of the product from the first PCR, again using a high-fidelity polymerase with the same increased elongation times/cycles as the first PCR.
8. Run the 2nd PCR product on a 2% agarose gel. If a single band can be seen and is at the appropriate molecular weight, despite possibly being quite faint or including high molecular weight smears, it can be sequenced.
9. Sequence PCR products that produce a visible band on the agarose gel. The same primer used in the 2nd PCR and upstream of the desired point mutations rather than upstream of the cut, can also be used for sequencing. To clean the DNA, we typically mixed 3 μL ExoSAP-IT with 7.5 μL of the PCR product, incubated at 37 °C for 5 min and 1 min at 80 °C, then sent the resulting mixture in for sequencing.
10. Guide RNA/donor DNA pairs that show visible integration of the desired mutations in more than 50% of the blastocysts are good candidates for making mice with these point mutations. The different pairs can also be compared to choose the best among them. If quantitation of the percent of indel and point mutations in each blastocyst is desired, Synthego’s ICE program works well.

### 3.3. CRISPR Component Toxicity

The overall workflow for testing first with blastocysts when engineering a mouse is shown in Figure 2. After making or buying Cas9 protein, the appropriate guide RNA, and donor DNA, these 3 components are mixed together and injected into zygotes (Figure 2, Step 1). Due to factors such as the quality of each component, not all zygotes may survive this step (Table 1). In our experiments, only the guide and donor were varied so it is apparent from Table 1 that for the *Magi1*, sgRNAs-M79R and –M67, and *Atox1*, sgRNA-A43 guide/donor pairs, one or both of these components are more detrimental to embryo health than for the remaining guide/donor pairs. However, the injection mixtures were sufficiently nontoxic to allow us to determine the integration efficiency in blastocysts of these guide/donor pairs and then use the best pair to make mice with the desired mutations.

### 3.4. CRISPR Component Leakage

Our experience is that, despite the large expected dilution assumed to be occurring in the zygote dish, changing micropipettes is not sufficient to prevent contamination of zygotes with donor DNA and Cas9/guide RNA complexes leaked from the first micropipette when injecting with a different guide/donor pair in a second micropipette. For this reason, each guide/donor pair was injected on a different day.

### 3.5. Repeatable Donor Integration Efficiency with the Same Guide/Donor Pair

**Figure 3:**
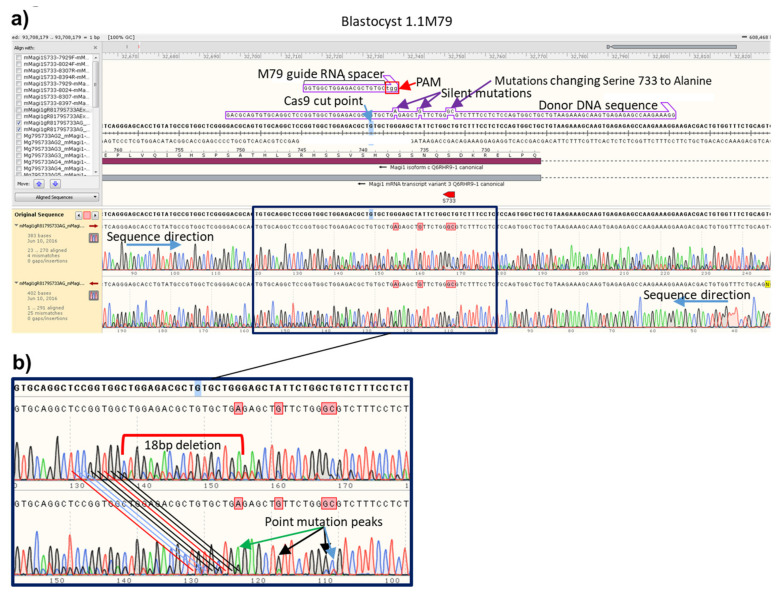
Example Sanger sequence chromatogram from a single mouse blastocyst after CRISPR treatment. Zygotes were injected with Cas9 protein, sgRNA (targeted to the *Magi1* gene), and donor DNA (to generate the point mutations leading to S733A, and containing silent mutations to alter the PAM sequence and improve incorporation of the desired mutations). The targeted region was sequenced and analyzed. (**a**) Chromatograms (http://www.snapgene.com/) from a single blastocyst (1.1M79) using primers in each direction and aligned to the WT sequence. The guide RNA spacer is the ~20 bp variable region of the guide that matches the DNA sequence adjacent to the PAM. Note the smaller peaks in the sequences after the Cas9 cut point. (**b**) The identity of the single peaks seen upstream of the Cas9 cut point can be matched to the smaller, extra peak seen after the cut point, shifted by 18bp in the reverse direction. These smaller peaks represent the 18 bp indel which existed in about 18% of the chromosome copies in this blastocyst. About 77% of this blastocyst’s chromosomes contained the desired point mutations. If the nucleotide indicated by the major peak in a Sanger sequence does not match the sequence it is being compared to, the SnapGene program used here to generate this image labels it in pink. In this case, the blastocyst’s sequences are being compared to the WT sequence.

**Figure 4:**
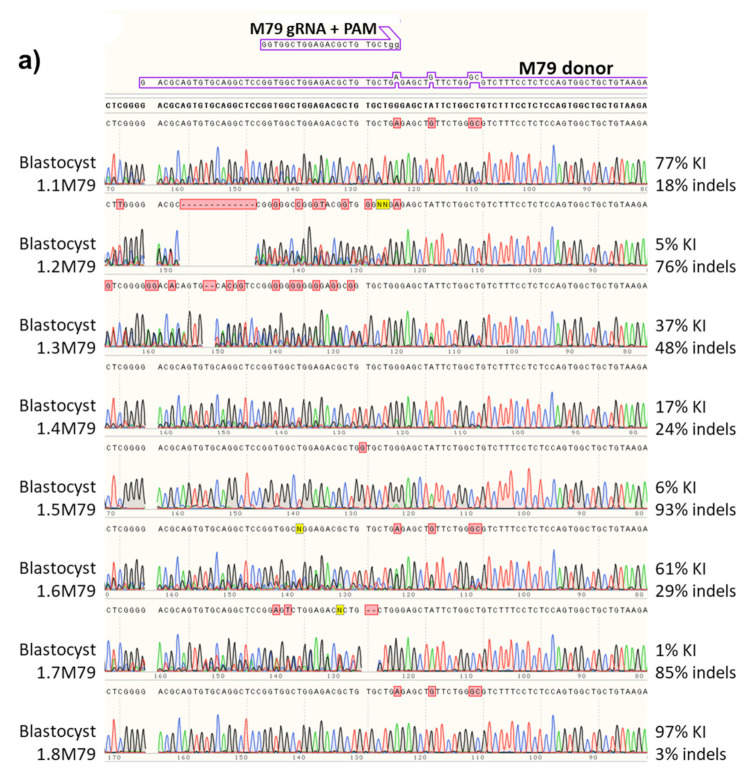

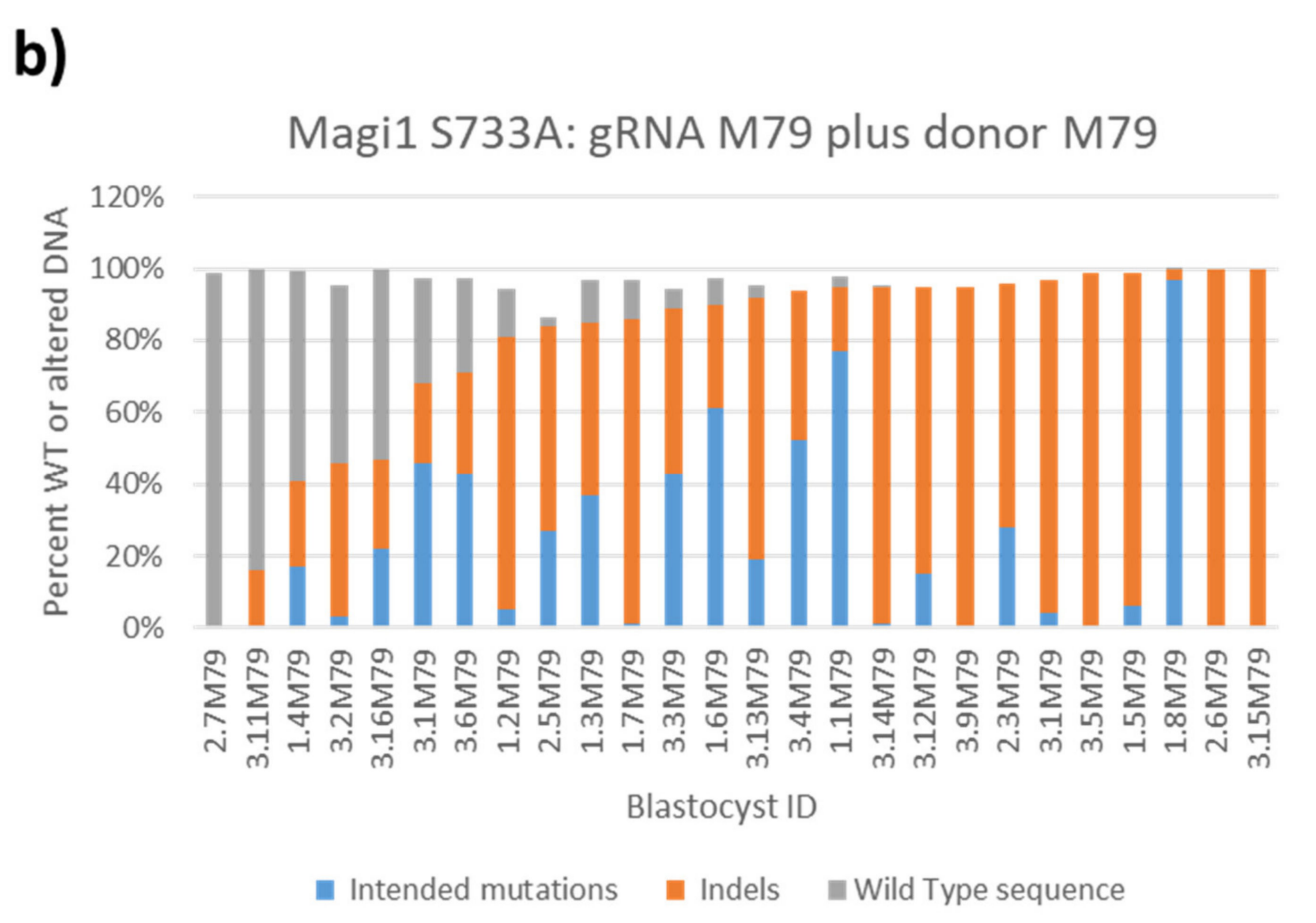
Silent mutations in the PAM region prevent recutting. The percentages of desired knock-in (KI) point mutations and indels in each blastocyst were quantified from their Sanger sequences using Synthego’s ICE program (https://ice.synthego.com/#/). (**a**) The chromatograms generated using the primer upstream of the S733A mutations, for 8 blastocysts injected with guide RNA gR8179 (M79), and the percent KI mutations and indels of each are quantified. The chromatogram as read from right to left of the blastocyst 1.1M79 from Figure 3 is in the top position. The sequence for each blastocyst is shown above its chromatogram. The WT sequence is shown in bold at the very top. (**b**) Bar graph of the percent KI mutations, indels and WT sequence quantified for each blastocyst from three separate experiments with guide M79. Each bar shows the quantified percent WT, indels and desired point mutations for a single blastocyst. The three experiments were combined and bars organized from blastocysts containing the least percent of cut genomic DNA to the most cut. Results from each experiment appear repeatable as data from each experiment show roughly the same pattern of percent indels and donor sequence integration. In addition, the fraction of DNA containing indels plus KI mutations does not exceed 100%. Once the altered PAM sequence is incorporated into the genomic DNA, the silent PAM point mutation appears to prevent additional editing by Cas9 and therefore prevent indels from forming in the same chromosome.

After 3 to 3.5 days of development (Figure 2, Step2), blastocysts and morulae were lysed, then assayed using nested PCR done at 60 cycles each, and the second PCR product Sanger sequenced (Figure 2, Step3). A typical example sequence from one blastocyst (1.1M79) treated with the *Magi1* sgRNA-M79/donor DNA pair (experiment 1M79) is shown using the SnapGene program in Figure 3. The donor is meant to change the mouse *Magi1* genomic DNA sequence of two bases, CT, to GC, altering serine 733 to alanine. Using Synthego’s ICE programs to calculate the percentage of indels (KO program) and desired point mutations (KI program), this blastocyst was found to contain 18% of one 18 base deletion, 77% of the desired point mutations, and 3% WT sequence. In the same way, the type and extent of mutations in the 7 other blastocysts (1.2M79 through 1.8M79) in the same 1M79 experiment were analyzed and quantified (Figure 4a). The injection of zygotes with this donor and guide pair was repeated on two more occasions (2M79 and 3M79). The percent indels, desired point mutations, and wild type sequence calculated from each resulting blastocyst from all three of these experiments is displayed together in a bar graph (Figure 4b), arranged from blastocysts containing the least percent of altered genomic DNA to the most. The results show that although the amount of cutting appears to vary quite a bit between each blastocyst in the same experiment, individual blastocyst data from the three experiments largely overlapped (Figure 4b, compare blastocyst results from 1M79, 2M79 and 3M79 with each other). Thus, when the same sgRNA-M79 guide and donor pair were used, roughly the same range of cutting occurred in the blastocysts overall between the three experiments done, 1M79, 2M79 and 3M79 (Table S4). This was true whether the Cas9 used was in the form of mRNA (1M79) or protein (2M79 and 3M79). Since the general consensus is that protein is better, we did the remaining experiments with Cas9 protein.

Three other guide/donor pairs were also designed to change mouse Magi1 serine 733 to alanine. Each was used in 2 or 3 blastocyst experiments. Blastocyst sequences resulting from using these pairs were also analyzed (Figure S1a–d). Again, despite variation in the percent of cutting occurring between blastocysts in the same experiment, the average amount of cutting for blastocysts in one experiment was roughly the same for blastocysts treated with the same guide/donor pair in the other experiments (Table S4).

We also noted that for all these guide/donor pairs, considering an inherent Sanger sequencing error rate in our ICE calculation of up to 8%, the percent of WT, indel and desired point mutation when added together for each blastocyst never went above 100% ± 8%. Given the relatively high cutting rate using these guides, this implies that the silent mutation in the PAM or guide RNA seed region sequence was effective in preventing additional editing of genomic DNA that had incorporated the silent mutation [9].

### 3.6. All-or-None Sequence Integration with Complementary Donor

**Figure 5:**
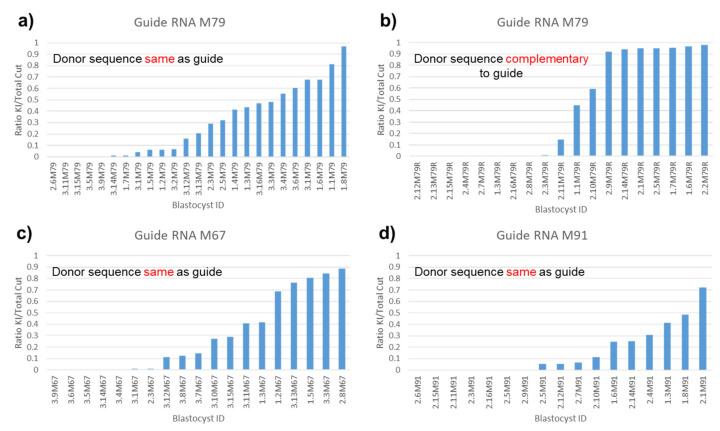
Same-sense donor sequence integrates randomly while complementary donor sequence integrates in a more all-or-none fashion, and silent mutations eliminate the strong distance bias. Data from experiments with Magi1 guide RNA M79, M67, and M91 and paired donor DNA treated blastocysts were bar graphed to study donor integration. For each guide/donor pair, data from two or three separate experiments were combined. Each bar shows the ratio of desired point mutation KIs per total cut genomic DNA for a single blastocyst. Bars are ordered from those with the lowest ratio of desired KI mutation per total cut to those with the highest ratio. (**a**) Blastocysts treated with guide RNA M79 and same-sense donor DNA. Desired mutation is 18 bp from cut. (**b**) Blastocysts treated with guide RNA M79 and complementary donor DNA. As with the same-sense, the desired mutation is 18 bp from cut. (**c**) Blastocysts treated with guide RNA M67 and same-sense donor DNA. Desired mutation is 31 bp from cut. (**d**) Blastocysts treated with guide RNA M91 and same-sense donor DNA. Desired mutation is 5 bp from cut. Both same-sense and complementary donors integrate their sequence into roughly half of the blastocysts with cut DNA but the pattern of integration is different. Those donors which are same-sense to the guide appear to integrate their sequence into a random proportion of the blastocyst’s cells, while the donor sequence that is complementary to the guide appears to be integrated more often into either all of the cells of the blastocyst or none of them. Furthermore, silent mutations eliminated the strong distance bias, possibly by preventing homologous recombination in the nonidentical region, forcing recombination to occur beyond it.

Of these 4 guide/donor pairs used to alter serine 733 to alanine, three had donors that contained the guide sequence while one had a donor that contained sequence complementary to the guide (Figure 5). It has been noted that there is a difference in the efficiency of sequence integration between a donor with sequence complementary to the guide and a donor with same sense as the guide [8]. To analyze the difference in the way the same-as-guide and complementary-to-guide sequence containing donors appeared to be incorporating their sequence into the genome, we regraphed the same data for each blastocyst to show the ratio of percent donor sequence integrated over percent total cut (i.e., leading to a change in sequence) where the total percent cut is assumed to be reflected by the sum of the percent indels plus percent desired point mutations (Figure 5). This assumption is based on our observation that each indel and integration of the desired point mutation seems to represent the only cutting event occurring in that chromosome, since additional editing of the altered chromosome does not appear to be happening to any detectable extent. By calculating the proportion of donor DNA sequence integrated from only the total cut DNA and not all the DNA in a blastocyst, the effect of the different efficiencies of cutting directed by different guides on donor sequence integration was eliminated. This allowed us to make clearer observations on how donors behaved based on the characteristics of the donor itself.

After arranging the data from the lowest ratio of integrated sequence to the highest, it became clear that the same sequence donors incorporated their sequence to a fairly random extent in different blastocysts (Figure 5a,c,d). In contrast, when compared to the same sequence donor using the same guide, the complementary donor appears mostly to be incorporating its sequence in an all-or-none fashion (Figure 5a vs. Figure 5b). One possible interpretation is that sequence incorporation of complementary donor happens much earlier during blastocyst development than for same sense donor.

### 3.7. Increased Knock-in Efficiency of Distant Mutagenesis Sites with Silent Mutations

We have observed that recombination can occur between the cut site and a desired mutation if the homology between the two is more than 10 bp long. When this happens, the intended mutations are not inserted into the genome. Moreover, if a guide’s PAM and seed region remain intact after HR, it can be re-targeted by the sgRNA/Cas9 complex after donor sequence integration [9,25]. To test whether proper placement of silent mutations can address these problems, we introduced silent mutations in the donor DNA approximately every 10 base pairs between the desired mutation and the guide’s PAM/seed region mutation. This allowed point mutations that are more than 10 bp away from the cut site to be efficiently integrated into the genomic DNA.

We tested four sgRNA/donor DNA pairs with a distance between their cut site and desired mutations of from 5 bp to 31 bp (Table S1, M67, M79, M79R, M91 paired donor DNA sequences, Figure 5). Mouse *Magi1* sgRNA M67 (Table S1, M67, Figure 5c) cuts 31 bp away from the desired mutations, and its paired donor has 6 fairly evenly spaced silent mutations between the PAM and the desired mutations. sgRNA M79 (Table S1, M79, M79R, Figure 5a,b) cuts 18 bp away and its paired donors (with either same-sense or complementary sequence to the sgRNA) have 2 silent mutations. sgRNA M91 (Table S1, M91, Figure 5d) cuts 5 bp away and has no silent mutations. Despite directing cutting beyond the usual 30 bp advised limit from the desired mutation, donors incorporated their sequence with about the same efficiency using guide M67 as for guide M79 and the same or slightly better than for guide M91, the guide closest to the desired point mutations (Figure 5a vs. Figure 5c vs. Figure 5d). For M67 sgRNA/donor DNA pair (desired mutation 31 bp to cut), 58% of embryos (11/19) had a donor sequence integration rate of >5% among the targeted alleles (total cut) as compared with 35% (6/17) of embryos in M91 sgRNA/donor DNA pair (5 bp to cut). Similarly, M79 pairs (18 bp to cut) had 56% (14/25, same-sense donor) and 58% (11/19, complementary donor) of embryos with an integration rate of >5% among targeted alleles (total cut). Taken together, these results indicate that introducing silent mutations every 10 or less bases between the cut site and desired mutagenesis site is an effective way to increase knock-in efficiency for mutations far from the cut site.

### 3.8. Similar Donor Integration in All Genes Tested

As was done for the mouse *Magi1* guide/donor pairs experiments, the effectiveness of the guide directed cutting and point mutation integration of several guide/donor pairs for two other genes, mouse MapK7 and Atox1, were also quantified and graphed (Figure S2). The donors for these genes were all same-sense as the guide sequence. Other than in the experiments for guide A27, similar rates of cutting were again seen between different experiments of the same guide/donor pair but, unlike *Magi1*, were widely different between different guide/donor pairs (Figure S2a–d, Table S4). In contrast, the graphed ratio of point mutation integration to total cut for the guide/donor pairs of these genes, as with the *Magi1* experiments, showed a much more similarly random distribution of donor sequence incorporation once the DNA was cut (Figure S2, compare a with b vs. e with f).

### 3.9. Unequal Efficiency in Dual Donor Sequence Integration

**Figure 6:**
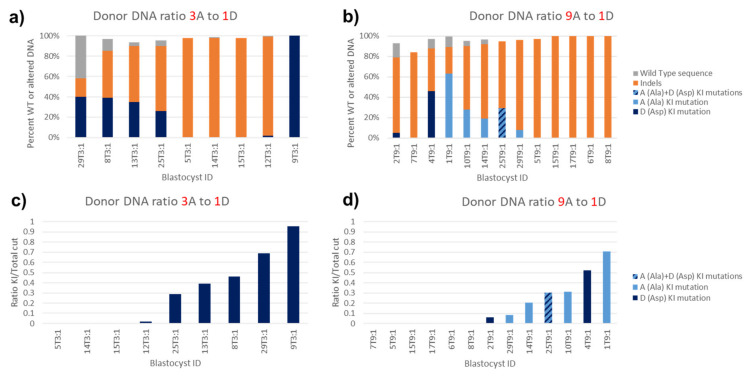
Sequence incorporation by two DNA donors (one for S202A and one for S202D) injected simultaneously. Simultaneous incorporation of two different KI sequences into the same genomic DNA position occurred when, along with Cas9 and a single guide RNA, zygotes were injected with a mixture of two donors. One donor had the sequence to convert mouse *Terf2ip* serine 202 into alanine (A) and the other, into aspartic acid (D). (**a**,**b**) The proportion of wild type, indel, A KI mutation and D KI mutation sequences were quantified and plotted. (**c**,**d**) The ratio of successful KI to total detected cut DNA was plotted. (**a**,**c**) A mixture of three times more donor DNAs directing for A than donor DNA directing for D was injected. Despite having 3 times more donor DNA available, no A KI mutations were seen, only D. (**b**,**d**) A mixture of nine times more donor DNAs directing for A than donor DNA directing for D was injected. Slightly more blastocysts incorporated the A donor sequence than did D. One blastocyst incorporated both the A and D donor sequences.

**Figure 7:**
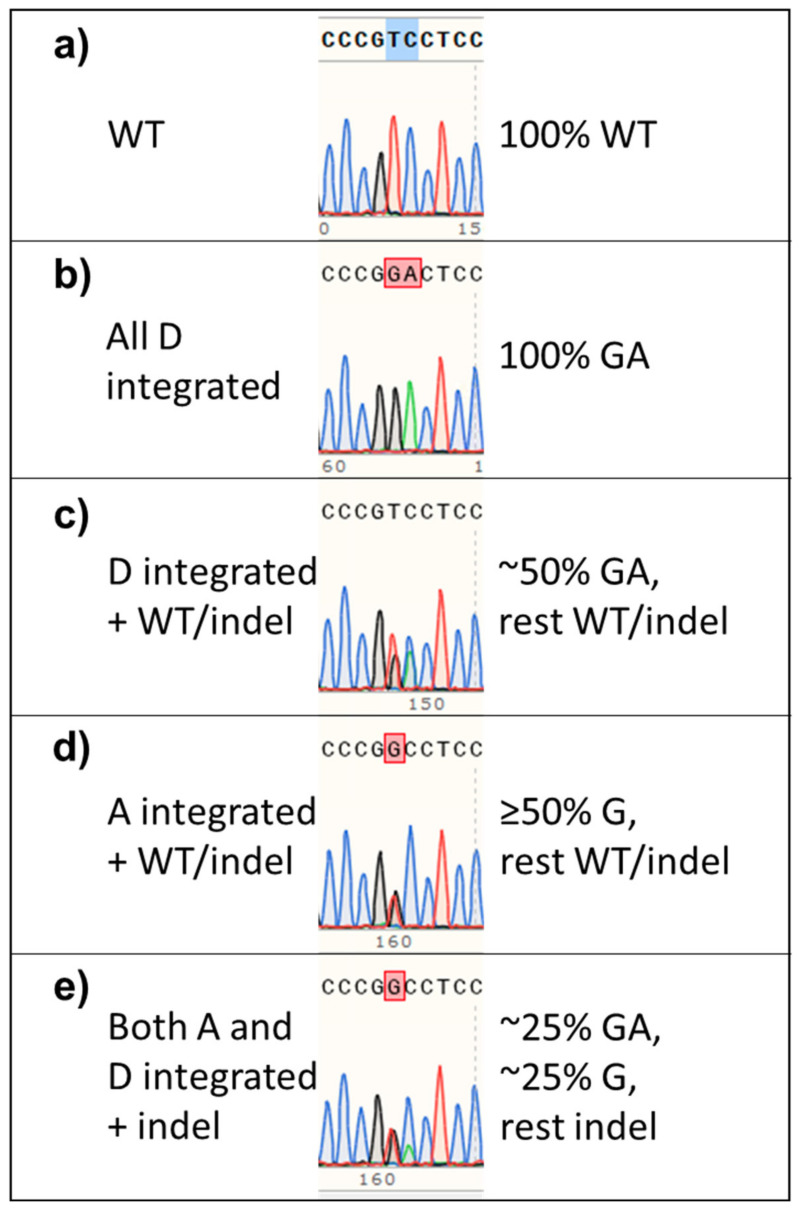
Sanger sequences of blastocysts integrating two different mutations into the same position on homologous/sister chromosomes. In most cases where two donors were simultaneously injected, either the D donor’s sequence or the A donor’s sequence was integrated. The WT sequence (**a**) was changed from TC to GA (two-point mutations, (**b**,**c**)) to produce the D amino acid and from T to G (one-point mutation, (**d**)) to produce the A amino acid. The two peaks for the D mutation are of a similar height, regardless of whether all (**b**) or some (**c**) of the bases replace the WT sequence. In one case, the two peaks of the integrated point mutations were of clearly different heights and the WT peaks at the same position were inversely of different heights (**e**) implying that both donor sequences were integrated into the genomic DNA in the same blastocyst. The sequence for each blastocyst is shown above its chromatogram. The WT sequence is shown at the very top.

Finally, using the *Terf2ip* gene, we tried to simultaneously incorporate two different point mutations into the same position on the blastocyst genome. Two donors, each containing point mutations to change the same codon, serine 202, one with DNA altered to make alanine and the other, aspartic acid, were mixed together with Cas9 and a guide RNA, and the mixture injected into each zygote. The percent of each point mutation, indel, and WT sequence per blastocyst was quantified and graphed (Figure 6). The ratio of the alanine (A) donor to the aspartic acid (D) donor was eventually increased to 9 to 1 since there appeared to be a strong preference to integrate the D donor sequence over that of the A donor (Figure 6a,c versus Figure 6b,d). At this donor ratio, 4 of the blastocysts had incorporated the amino acid point mutation sequences (Figure 7a WT vs. Figure 7b–e), one of which had integrated the point mutation for A and two, the mutations for D, while one blastocyst harbored genomic DNA coding for both A and D (Figure 6b,d and Figure 7e). The values obtained from the ICE KI analysis for the percent of A donor sequence and D donor sequence in the one blastocyst which had both A and D point mutations were identical, implying that equal amounts of each donor were integrated into the genomic DNA of that blastocyst.

### 3.10. Similar Donor Sequence Integration Efficiency in Mice vs. Blastocysts

For each gene, the most efficient or one of the most efficient non-complementary guide/donor pairs was usually used to make mice. The difficulty in generating the appropriate mice corresponded in general to that of getting the correct integration for a guide/donor pair as found in the blastocysts, with *Atox1* (A27) requiring the greatest number of pups and *Magi1* (M79) the least (Table 2).

## 4. Discussion

Recent advances in the CRISPR/Cas technology have made genetic engineering of mice and other vertebrates significantly more efficient and less labor-intensive than other more traditional methods. In large part, the success of the CRISPR/Cas method in making precise changes in the genomic DNA depends on the combined action of guide RNAs and donor DNAs, which cannot be assessed by currently available in vitro and in silico testing. In this study, we developed a protocol in which mouse blastocysts were used to rapidly and cheaply determine the ability of a CRISPR guide RNA and donor DNA pair to produce a desired genomic sequence prior to using them for engineering mice. This was done by Sanger sequencing DNA from blastocysts that were previously injected with the same CRISPR components intended to produce the engineered mice. The protocol should also be applicable and appropriate for pretesting any mouse genetic engineering technique used to generate precise point mutations such as base editing [29] and prime editing [30]. In addition, our study provided us with clues on how different donor DNAs behave.

### 4.1. Advantages of the Blastocyst Protocol

Mouse zygotes were injected with CRISPR components containing Cas9, guide RNA and donor DNA intended to produce point mutations, and allowed to develop to the blastocyst stage. Total DNA from the roughly 200 cells in each blastocyst was isolated, and by using a slightly altered nested PCR protocol, we were able to obtain clearly readable Sanger sequences. These sequences were then used to determine which guide/donor pairs are better at integrating the desired sequence into the mouse genome. These procedures are fast, eliminating the weeks of gestation and postpartum needed to make the same determination by genotyping mouse pups.

Our results suggest that as far as the CRISPR/Cas9 system goes, it is sufficient to read the Sanger sequences and verify that at least 50% of blastocysts carry the desired mutation. Factors coming into play beyond blastocyst formation, such as the effect of the mutated gene on mouse development and viability, the state of the surrogate mother, and whether the desired CRISPR mutation resides in the germ line versus somatic cells, can all affect how many mice with the apparently correct mutation are actually born. Success with blastocysts may not indicate in turn that the same success will occur in obtaining viable pups with the desired mutation. However, if the CRISPR components work well in the blastocyst, one can concentrate on looking for the cause of failure elsewhere rather than focusing on whether the CRISPR step was correctly carried out or not.

If zygotes fail to develop to the blastocyst stage, this technique provides a quick way to determine which, if any, of the injection mixture components might be causing embryonic lethality. By removing a suspected component(s) and checking blastocyst development, one can quickly determine whether and which of the components cause toxicity. If the impact of the CRISPR mixture is not too severe, reduced viability can be compensated for by increasing the number of zygotes injected. Because only the guide and donor were varied in the experiments described above, the effect of each guide/donor pair on embryo viability could be compared (Table 1).

In this protocol, one μL of the ~7–10 μL blastocyst lysate was used to quickly amplify the region of interest with nested PCR. Others have used whole genome amplification (WGA) to amplify blastocyst DNA. Though a more expensive and time-consuming approach, WGA has the added benefit that one is looking at the entire range of CRISPR affected DNA. However, our fast, inexpensive method was fully adequate for determining guide/donor efficiency for use in mice.

Making controlled transgenic changes through CRISPR in the DNA of blastocysts of organisms such as the mouse has several added advantages over that of typical tissue culture cells. The success rate of integrating point mutation(s), or inserting tags or other larger pieces of DNA is greatly improved by direct injection or electroporation of CRISPR components into the zygote nucleus and by the absence of G1 in the cell cycle of the embryo during early development [31,32]. Since the cell immediately goes from mitosis into S phase, the level of HDR proteins remains relatively high, allowing for efficient incorporation of donor DNA sequence into the genomic DNA near the sgRNA induced cut point. Tissue culture cells, on the other hand, spend a large portion of their time in G1 during which double strand breaks are repaired by nonhomologous end joining to a much greater extent than by HDR [33].

### 4.2. Reproducibility of Blastocyst Protocol

Since this blastocyst protocol was designed to predict success in making CRISPR engineered point mutations in mice, its repeatability was assessed by injecting the same Cas9/gRNA/donor solutions on different days, although the injectionist and equipment used did not vary. To quantify the Sanger sequencing results, the Synthego ICE program was chosen for its ease of use and reliability. The results obtained by ICE quantification showed that, although both the extent of cutting and donor sequence integration varied widely between blastocysts in the same experiment, the random range of each usually overlapped fairly consistently between different experiments using the same gRNA/donor pair. For the Atox1 guide/donor pair A27 which was predicted to cut efficiently, we performed only two tests, and the blastocyst results from the two experiments barely overlapped (Figure S2c, Table S4), suggesting that there was a problem with the CRISPR system for one of the experiments. For this reason, we recommend checking the CRISPR components and repeating the blastocyst test several times if a guide/donor pair unexpectedly gives poor results.

The degree of cutting leading to indels and donor DNA integration did correlate with the predicted success from the CRISPOR program although sometimes not strongly. Other factors such as the method used to generate the guide RNA [34], technical expertise of the injectionist, or level of contaminants such as endotoxins or RNase in the CRISPR component mixture can also affect the outcome [2,5]. However, as long as a reasonable percentage of correctly engineered blastocysts are obtained, it is not necessary to troubleshoot.

### 4.3. Similar Pattern of Donor Integration into Different Genes

Although the extent of cutting varied widely between many of the different gRNAs, the pattern and degree of donor sequence incorporation into the already cut DNA was surprisingly similar between donors of the type in which the sequence was the same as the gRNA (compare how graphs a-d in Figures S1 and S2 differ widely between each other while graphs e-h in Figures S1 and S2 do not differ nearly as much). The frequency of cutting is dependent on the purity and concentration of guide and Cas9 injected, as well as the cutting efficiency dictated by the guide sequence, all of which can be highly variable between different guides and experiments. On the other hand, the presence of intervening silent mutations in the donor DNA seems to abrogate the distance-from-cut effect so once the genomic DNA is cut, integration of these similar length donors depends only on the more uniform mixture of repair components generated by the cell. This may be what leads to the similar patterns of integration.

### 4.4. Increased Integration Efficiency Using Silent Mutations

It is known that as the distance between a mutation and a cut site increases, the efficiency with which the mutation integrates into the genomic DNA declines [9,10]. This means recombination is occurring more frequently the closer to the cut it is and is why the further away a desired mutation is from the cut, the lower the chance that it will be incorporated into the genomic DNA. The mutation(s) furthest from the cut is usually the desired one. We have found that by having a silent mutation roughly every 10 bp or less between the cut site and the desired mutation site, the integration efficiency of the furthest point mutation can be dramatically increased. Because of this, we typically integrated silent mutations about every 10 bp between the cut site and the desired mutation site.

Using this technique, we found that the rate at which this furthest mutation integrates appears to be similar to that of donors with desired mutations at or near the cut. For the donor with the longest distance between cut and desired mutations, 31 bp, we were forced to replace codons every 5 or 6 base pairs in order to make the overall concentration of tRNAs used by the new codons be similar to that of the original codons (see Section 2.3 “Design and Preparation of Single Strand Donor DNA” in Materials and Methods). This donor worked very efficiently, as or more efficiently than one containing no silent mutations and the desired mutations 5 bases from the cut site (Figure 5c vs. Figure 5d).

The genomic DNA must recombine with or start copying from the donor DNA in order to incorporate the donor sequence, and it is not entirely clear how a distance of around 10 or less bases between mutations in the mutated region prevents this from happening. It may be that the temperature of annealing between donor and genomic DNA is reduced enough to prevent sufficient binding for recombination/copy initiation to occur. The Rad51 bound 3′ end of cut and resected DNA produced during homologous recombination rapidly interrogates double stranded DNA using a length-based recognition mechanism in which it searches for tracts of microhomology of at least 8-nucleotides [35]. Accordingly, any shorter homology and the searching single stranded end will not stick. This may explain why the donor with multiple silent mutations 5 to 6 bp apart in the 31 bp region between cut and desired mutations worked so well. By having a region in the donor stretching from the desired mutation to the cut, with closely spaced silent mutations, it is difficult for the searching cut and resected free end of the genomic DNA on the near side of the desired mutation to stick to the donor. Only the end on the far side of the cut can stick tightly to the donor and that end will copy all the donor’s mutations. By putting mutations close together between the cut and the desired mutation, recombination is forced to occur beyond the desired mutation. However, before using this technique to integrate desired point mutations far from the cut, one must still balance the possibility that a silent mutation may not really be silent [36] with the increased ease of getting a point mutation in far from the cut site.

### 4.5. Effect of Donor Direction and Number on Donor Sequence Integration

**Figure 8:**
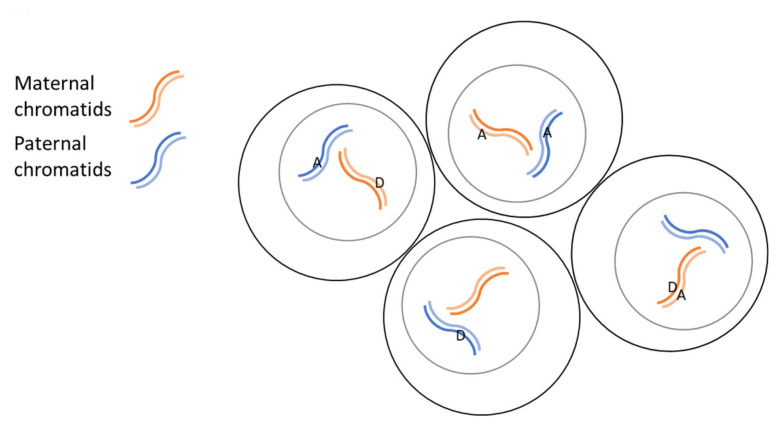
Some examples of possible genome alterations after simultaneous treatment with two donors. One donor contains the A mutation and the other donor, the D mutation. The A and D mutations are shown randomly incorporated into different sites of duplicated homologous chromosomes. Note that in each cell, there are 4 sites to which A and D mutations can be incorporated. Thus, many other combinations of A and D integration into a single cell are also possible, as are different combination of these cells in a single blastocyst or mouse. However, in our experiments shown in Figure 7, most of the blastocysts and mice contained either the A or the D alteration. Only one pair of paternal (blue) and maternal (red) chromosomes out of the total 20 chromosomes in a mouse cell is shown in this figure.

In addition to asking whether CRISPR data obtained from our new blastocyst protocol could be used to predict success in making CRISPR engineered point mutations in mice, we attempted to answer two other questions: (1) Does a donor containing sequence complementary to the variable region of the guide RNA work better, the same as or worse at correctly altering the genomic sequence than donor containing the same sense sequence? (2) Is it possible to use two donors simultaneously, identical in sequence except for different mutations at the same position on each, to integrate into different copies of the same (homologous or sister) chromosome in a blastocyst or mouse (Figure 8)?

In answer to question (1), donor containing sequence completely complementary to the guide RNA appears to integrate into the cut DNA in about the same percentage of blastocysts as donor containing the same sequence as the guide, but the pattern of integration within the blastocyst itself was different. When donor with complementary sequence was used, most blastocysts appeared to have either integrated the mutations into the cut DNA of all of its cells or the cut DNA of none of its cells. Blastocysts treated with same-sense-as-guide donor, on the other hand, had randomly different proportions of their cells with the point mutations integrated. The simplest explanation is that integration into the cut DNA happens very early for the complementary donor and at various later random times during blastocyst development for the same-sense donors. Based on their results, Richardson et al. developed a model in which the end of the same-sense-as-guide strand on the non-PAM side of the cut genomic DNA is released from Cas9 much earlier than the other three cut strand ends [8]. Their data suggests that this released strand is available to bind to complementary donor ssDNA well before the other cut ends become accessible to the donor and repair systems [37]. This model could explain why the complementary-to-guide donor in our experiments appears to integrate much sooner than the same-sense-to-guide donors. A complementary donor will bind to this released strand soon after Cas9 cuts and be immediately available for sequence incorporation [38,39] when Cas9 eventually falls off [40,41]. On the other hand, after cut genomic DNA is freed from Cas9, it would take time for the same-sense-as donor to find the now available genomic free ends, time during which alternative repair processes, either perfect or imperfect repair, could take place. Perfect repair could allow time for further cell divisions and rebinding of the Cas9 complex before same-sense-as donor sequence incorporation finally occurred.

The region where the donor DNA is complementary to the guide’s variable region must be a perfect match for the above scenario to work well. If silent mutations are added in the 20 base variable region complementary to the guide RNA, this is expected to abrogate the early incorporation of the donor sequence that we postulate is happening. We would expect that the binding of the donor DNA which happens when the first Cas9 cut strand of genomic DNA is released, would no longer occur efficiently since the match would no longer be perfect. We postulate that this is the scenario occurring with the partially complementary match to the guide RNA sequence for the Terf2ip donors. The donors have two mutations spaced 9 bp apart in the sequence complementary to the guide’s 20 base variable region, one of which is in its seed region (Table S1).

Question 2 arose because we were interested in seeing what phenotypes result from mutating mouse Terf2ip serine 202 into one of two other amino acids. One mutation converts the serine into alanine (A) which is intended to constitutively mimic the serine non-phosphorylated state while the other mutation, into aspartic acid (D), to mimic the phosphorylated state of the serine. In order to save on costs and time, two different donors, one coding for the constitutively phosphorylated amino acid mimic and the other, the non-phosphorylated mimic, were injected simultaneously into the same zygote. The blastocyst genome was then checked to see what the likelihood was of getting mice with both mutations at once. Examples of possible outcomes are shown in Figure 8.

The different donor sequences were found to successfully integrate into the same location on homologous/sister chromosomes in the same blastocyst. However, there was a 9 to 1 sequence integration preference for the D donor over the A donor. There are many possible factors that could explain this disparity including the fact that because of the additional point mutation in the D donor, one of the strong hairpins found in the A donor no longer forms. If using multiple donors simultaneously, our results suggest it is best to determine the optimal donor/donor ratio by using a technique like blastocyst sequencing, before using the donor mixture to make mice. The results also show that each mutation is not necessarily found in the same animal but if occurring in the same animal, that animal could be crossed to separate the two mutations into different mouse lines. This approach could cut costs in half by initially doing only one CRISPR project rather than two to get the mouse lines with each mutation.

## 5. Conclusions

The need for a clear, detailed protocol using blastocysts to test guide RNA cutting and donor DNA integration efficiencies led us to develop a simple, fast method and present it here in a step-by-step fashion. Quantifying data from CRISPR experiments on blastocysts not only allowed us to save substantial time and money by determining which guide/donor pair would best lead to successful sequence integration but also showed us that blastocyst DNA sequencing is itself useful as a tool for studying the effects of CRISPR on the genome.

## Figures and Tables

**Table 1:** Testing for sgRNA and donor DNA solution toxicity**.** Various sgRNAs and their paired DNA donors were tested for the three projects, *Magi1*, *Mapk7* and *Atox1*. The results indicate that for *Magi1*, sgRNAs-M79R and –M67, and for *Atox1*, sgRNA-A43, the guide and/or donor solutions were detrimental to embryo progression. Total: total number of zygotes injected; Blasts: blastocysts; DNP: did not progress to the late morula/blastocyst stage.

Project	sgRNA/ Donor Pair	Total	Number Morulae	Number Blasts	Number DNP	Number Dead	% Morulae	% Blasts	% Combined	% DNP	% Dead
Magi 1	M67	44	13	11	15	5	30%	25%	55%	34%	11%
	M79	45	6	25	13	1	13%	56%	69%	29%	2%
	M79R	51	7	15	23	6	14%	29%	43%	45%	12%
	M91	41	7	19	13	2	17%	46%	63%	32%	5%
Mapk7	E84	48	3	22	19	4	6%	46%	52%	40%	8%
	E87	51	10	23	17	1	20%	45%	65%	33%	2%
Atox1	A27	22	6	5	10	1	27%	23%	50%	45%	5%
	A43	19	3	6	6	4	16%	32%	47%	32%	21%

**Table 2:** Comparison of the percentages of desired mutation integration between blastocyst and mouse. Guide/donor pairs giving the better or best rate of desired mutation in blastocysts were usually used to make mice. For each gene being altered, the percentage of pups born with the desired mutation was compared to the percentage of blastocysts with the same mutation. A roughly similar pattern of insertional frequency was found between mice and blastocysts for the corresponding altered gene.

Project Designation	Gene (Guide RNA Used)	Mouse Designation	Pups with Desired Mutation/All Pups	Percentage F0 Pups with Desired Mutation	Percentage Blasts with Desired Mutation
GEMF-748	mMagi1 (M79)	Magi1-S733A-KI	5/7	71%	54%
GEMF-374	mMapK7 (E84)	Erk5-S496A-KI	2/9	22%	39%
GEMF-495	mAtox1 (A27)	Atox1-K3R-KI	4/20	20%	13%
GEMF-1128	mTerf2ip (T77)	Terf2ip-S202A-KI	4/18	22%	38%

## Supplementary Materials

The following are available online at https://www.mdpi.com/2073-4425/11/6/628/s1, Figure S1: Pattern of guide RNA cutting compared to pattern of donor DNA integration for different guide/donor pairs in Magi1., Figure S2: Different patterns of cutting, similar pattern of integration from different guide/donor pairs in different genes, Table S1: Sequence of single guide RNA and donor DNA pairs, Table S2: CRISPR component concentrations for each experimental condition, Table S3: Nested PCR primers used for sequencing, Table S4: Estimation of guide RNA directed cutting efficiency.

## Abbreviations

Blasts	Blastocysts
Cas9	CRISPR associated protein 9
CRISPR	Clustered Regularly Interspaced Short Palindromic Repeats
DNP	did not progress to the late morula/blastocyst stage
ICE	Inference of CRISPR Edits
KI	Knock-in
KO	knock-out
kb	kilobase
Tm	melting temperature
oligos	oligonucleotides
HDR	homology directed repair
ssDNA	single strand DNA
sgRNA	single guide RNA
WGA	whole genome amplification
WT	wildtype

## References

[1] Behringer R., Gertsenstein M., Nagy K.V., Nagy A. (2014). Manipulating the Mouse Embryo: A Laboratory Manual.

[2] Xu H., Xiao T., Chen C.-H., Li W., Meyer C.A., Wu Q., Wu D., Cong L., Zhang F., Liu J.S. (2015). Sequence determinants of improved CRISPR sgRNA design. Genome Res..

[3] Mehravar M., Shirazi A., Mehrazar M.M., Nazari M. (2019). In Vitro Pre-validation of Gene Editing by CRISPR/Cas9 Ribonucleoprotein. Avicenna J. Med. Biotechnol..

[4] Anders C., Jinek M. (2014). In vitro Enzymology of Cas9. Methods Enzymol..

[5] Horlbeck M.A., Witkowsky L.B., Guglielmi B., Replogle J.M., Gilbert L.A., Villalta J.E., Torigoe S.E., Tjian R., Weissman J.S. (2016). Nucleosomes impede Cas9 access to DNA in vivo and in vitro. eLife.

[6] Richardson C.D., Kazane K.R., Feng S.J., Bray N.L., Schaefer A.J., Floor S., Corn J. (2017). CRISPR-Cas9 Genome Editing In Human Cells Works Via The Fanconi Anemia Pathway. bioRxiv.

[7] Liang X., Potter J., Kumar S., Ravinder N., Chesnut J.D. (2017). Enhanced CRISPR/Cas9-mediated precise genome editing by improved design and delivery of gRNA, Cas9 nuclease, and donor DNA. J. Biotechnol..

[8] Richardson C.D., Ray G.J., DeWitt M.A., Curie G.L., Corn J.E. (2016). Enhancing homology-directed genome editing by catalytically active and inactive CRISPR-Cas9 using asymmetric donor DNA. Nat. Biotechnol..

[9] Paquet D., Kwart D., Chen A., Sproul A., Jacob S., Teo S., Olsen K.M., Gregg A., Noggle S., Tessier-Lavigne M. (2016). Efficient introduction of specific homozygous and heterozygous mutations using CRISPR/Cas9. Nature.

[10] Elliott B., Richardson C., Winderbaum J., Nickoloff J.A., Jasin M. (1998). Gene Conversion Tracts from Double-Strand Break Repair in Mammalian Cells. Mol. Cell. Biol..

[11] Lin S., Staahl B.T., Alla R.K., Doudna J.A. (2014). Enhanced homology-directed human genome engineering by controlled timing of CRISPR/Cas9 delivery. eLife.

[12] Song F., Stieger K. (2017). Optimizing the DNA Donor Template for Homology-Directed Repair of Double-Strand Breaks. Mol. Ther. Nucleic Acids.

[13] Fujii W., Kawasaki K., Sugiura K., Naito K. (2013). Efficient generation of large-scale genome-modified mice using gRNA and CAS9 endonuclease. Nucleic Acids Res..

[14] Sakurai T., Watanabe S., Kamiyoshi A., Sato M., Shindo T. (2014). A single blastocyst assay optimized for detecting CRISPR/Cas9 system-induced indel mutations in mice. BMC Biotechnol..

[15] Chu V.T., Weber T., Graf R., Sommermann T., Petsch K., Sack U., Volchkov P., Rajewsky K., Kühn R. (2016). Efficient generation of Rosa26 knock-in mice using CRISPR/Cas9 in C57BL/6 zygotes. BMC Biotechnol..

[16] Yao X., Zhang M., Wang X., Ying W., Hu X., Dai P., Meng F., Shi L., Sun Y., Yao N. (2018). Tild-CRISPR Allows for Efficient and Precise Gene Knockin in Mouse and Human Cells. Dev. Cell.

[17] Abe J., Ko K.A., Kotla S., Wang Y., Paez-Mayorga J., Shin I.J., Imanishi M., Vu H.T., Tao Y., Leiva-Juarez M.M. (2019). MAGI1 as a link between endothelial activation and ER stress drives atherosclerosis. JCI Insight.

[18] Shin I., Won J.H., Ko K.A., Shin J.-H., McBeath E., Thomas T., Giancursio C., RA Q.-Q., Taunton J., Hosokawa H. (2016). Abstract 8: The Membrane-associated Guanylate Kinase Ww and Pdz Domain-containing Protein 1 magi1 is Required for Disturbed Flow-induced Endothelial Inflammation and Atherosclerotic Plaque Formation. Arterioscler. Thromb. Vasc. Biol..

[19] Le N.-T., Takei Y., Izawa-Ishizawa Y., Heo K.-S., Lee H., Smrcka A.V., Miller B.L., Ko K.A., Ture S., Morrell C. (2014). Identification of Activators of ERK5 Transcriptional Activity by High-Throughput Screening and the Role of Endothelial ERK5 in Vasoprotective Effects Induced by Statins and Antimalarial Agents. J. Immunol..

[20] Chen G.-F., Sudhahar V., Youn S.-W., Das A., Cho J., Kamiya T., Urao N., McKinney R.D., Surenkhuu B., Hamakubo T. (2015). Copper Transport Protein Antioxidant-1 Promotes Inflammatory Neovascularization via Chaperone and Transcription Factor Function. Sci. Rep..

[21] Kotla S., Vu H.T., Ko K.A., Wang Y., Imanishi M., Heo K.-S., Fujii Y., Thomas T.N., Gi Y.J., Mazhar H. (2019). Endothelial senescence is induced by phosphorylation and nuclear export of telomeric repeat binding factor 2–interacting protein. JCI Insight.

[22] Addgene: CRISPR Guide. https://www.addgene.org/crispr/guide/.

[23] Concordet J.-P., Haeussler M. (2018). CRISPOR: intuitive guide selection for CRISPR/Cas9 genome editing experiments and screens. Nucleic Acids Res..

[24] Paix A., Folkmann A., Goldman D.H., Kulaga H., Grzelak M.J., Rasoloson D., Paidemarry S., Green R., Reed R.R., Seydoux G. (2017). Precision genome editing using synthesis-dependent repair of Cas9-induced DNA breaks. Proc. Natl. Acad. Sci. USA.

[25] Jinek M., Chylinski K., Fonfara I., Hauer M., Doudna J.A., Charpentier E. (2012). A Programmable Dual-RNA–Guided DNA Endonuclease in Adaptive Bacterial Immunity. Science.

[26] Luo C., Zuñiga J., Edison E., Palla S., Dong W., Parker-Thornburg J. (2011). Superovulation Strategies for 6 Commonly Used Mouse Strains. J. Am. Assoc. Lab. Anim. Sci. JAALAS.

[27] Kluesner M.G., Nedveck D.A., Lahr W.S., Garbe J.R., Abrahante J.E., Webber B.R., Moriarity B.S. (2018). EditR: A Method to Quantify Base Editing from Sanger Sequencing. CRISPR J..

[28] Norton W.B., Scavizzi F., Smith C.N., Dong W., Raspa M., Parker-Thornburg J.V. (2016). Refinements for embryo implantation surgery in the mouse: comparison of injectable and inhalant anesthesias—Tribromoethanol, ketamine and isoflurane—On pregnancy and pup survival. Lab. Anim..

[29] Lee H.K., Smith H.E., Liu C., Willi M., Hennighausen L. (2020). Cytosine base editor 4 but not adenine base editor generates off-target mutations in mouse embryos. Commun. Biol..

[30] Liu Y., Li X., He S., Huang S., Li C., Chen Y., Liu Z., Huang X., Wang X. (2020). Efficient generation of mouse models with the prime editing system. Cell Discov..

[31] King R.W., Jackson P.K., Kirschner M.W. (1994). Mitosis in transition. Cell.

[32] Ogura Y., Sasakura Y. (2017). Emerging mechanisms regulating mitotic synchrony during animal embryogenesis. Dev. Growth Differ..

[33] Cooper G.M. (2000). The Eukaryotic Cell Cycle. The Cell: A Molecular Approach.

[34] Wienert B., Shin J., Zelin E., Pestal K., Corn J.E. (2018). In vitro—transcribed guide RNAs trigger an innate immune response via the RIG-I pathway. PLOS Biol..

[35] Qi Z., Redding S., Lee J.Y., Gibb B., Kwon Y., Niu H., Gaines W.A., Sung P., Greene E.C. (2015). DNA Sequence Alignment by Microhomology Sampling during Homologous Recombination. Cell.

[36] Sharma Y., Miladi M., Dukare S., Boulay K., Caudron-Herger M., Groß M., Backofen R., Diederichs S. (2019). A pan-cancer analysis of synonymous mutations. Nat. Commun..

[37] Brinkman E.K., Chen T., de Haas M., Holland H.A., Akhtar W., van Steensel B. (2018). Kinetics and Fidelity of the Repair of Cas9-Induced Double-Strand DNA Breaks. Mol. Cell.

[38] Ma M., Zhuang F., Hu X., Wang B., Wen X.-Z., Ji J.-F., Xi J.J. (2017). Efficient generation of mice carrying homozygous double-floxp alleles using the Cas9-Avidin/Biotin-donor DNA system. Cell Res..

[39] Aird E.J., Lovendahl K.N., Martin A.S., Harris R.S., Gordon W.R. (2018). Increasing Cas9-mediated homology-directed repair efficiency through covalent tethering of DNA repair template. Commun. Biol..

[40] Jones D.L., Leroy P., Unoson C., Fange D., Ćurić V., Lawson M.J., Elf J. (2017). Kinetics of dCas9 target search in Escherichia coli. Science.

[41] Ma H., Tu L.-C., Naseri A., Huisman M., Zhang S., Grunwald D., Pederson T. (2016). CRISPR-Cas9 nuclear dynamics and target recognition in living cells. J. Cell Biol..

