# Supplementary material for: Rapid Evaluation of CRISPR Guides and Donors for Engineering Mice
Source: Genes (Basel). 2020 Jun 8;11(6):628. doi: 10.3390/genes11060628 (PMC7349695; doi:10.3390/genes11060628)
Supplement: Supplementary file 1 [file genes-11-00628-s001.pdf]

genes

MDPI

Supplementary Materials

Rapid Evaluation of CRISPR Guides and Donors for Engineering Mice

Elena McBeath [1,*], Jan Parker-Thornburg [2,*], Yuka Fujii [3,†], Neeraj Aryal [2], Chad Smith [2], Marie-Claude Hofmann [1], Jun-ichi Abe [3] and Keigi Fujiwara [3]

[1] Department of Endocrine Neoplasia and Hormonal Disorders, University of Texas MD Anderson Cancer Center, Houston TX 77030
[2] Department of Genetics, University of Texas MD Anderson Cancer Center, Houston TX 77030,
[3] Department of Cardiology, University of Texas MD Anderson Cancer Center, Houston TX 77030
† Present address: Department of Radiology, Houston Methodist Research Institute, Houston TX 77030

Table S1. ***Sequence of single guide RNA and donor DNA pairs.*** The ten different guide RNA and donor pairs used in this study are listed. The left column indicates the designation used for each guide/donor pair, with M referring to the *Magi1* gene, E to the *Mapk7* gene, A to the *Atox1* gene and T to the *Tirf2ip* gene. The numbers that follow refer to different cut sites on the gene. The middle column shows the sequence of the variable region of the guide RNA (as RNA, contains U instead of T) in uppercase letters and the PAM in lower case, and the right column shows the sequence of the donor DNA used with that guide. Silent mutations are shown in bolded blue and purple, purple being in the PAM and guide seed regions, and desired mutations in bolded red.

	**RNA guide variable sequence + PAM**	**Donor DNA sequence**
**M67**	AGCACAGCGTC<br>TCCAGCCACcgg	GGCTCTCTCACTTGCTTTCTTACAGCAGCCACTGGAGAGGAAAGA**CG**CCCAGAA**C**<br>AGCTC**T**CAGCA**T**AGCGT**G**TCCAG**T**CACCG**C**AGCCTGCACACTGCGTCCCCGAGCC
**M79**	GGTGGCTGGAG<br>ACGCTGTGCtgg	GACGCAGTGTGCAGGCTCCGGTGGCTGGAGACGCTGTGCTG**A**GAGCT**G**TTCTGG**G**<br>**C**GTCTTTCCTCTCCAGTGGCTGCTGTAAGAAAGCAAGTGAGAGAGCCAAGAAAGG
**M79R**	GGTGGCTGGAG<br>ACGCTGTGCtgg	CCTTTCTTGGCTCTCTCACTTGCTTTCTTACAGCAGCCACTGGAGAGGAAAGA**CG**C<br>CCAGAA**C**AGCTC**T**CAGCACAGCGTCTCCAGCCACCGGAGCCTGCACACTGCGTC
**M91**	GCTGTGCTGGG<br>AGCTATTCtgg	GACGCAGTGTGCAGGCTCCGGTGGCTGGAGACGCTGTGCTGGGAGCTATTCTG**AG**<br>**C**GTCTTTCCTCTCCAGTGGCTGCTGTAAGAAAGCAAGTGAGAGAGCCAAGAAAGG
**E84**	CGAGGCTCAGG<br>CGCCTCCAAggg	CTGGCGTTCCTGAGCTGTCACGGGCTTTCGAGGCTCAGGCGCCTCCA**G**GGGTGCA<br>**GC**GGGCCCATCTGCAGAAAAGTTGGACAAGGGAAAGGTAATGGCTTACAGATCT<br>G
**E87**	TGGGCCCAGTG<br>CACCCTTGGagg	CAGATCTGTAAGCCATTACCTTTCCCTTGTCCAACTTTTCTGCAGATGGGCCC**GC**TG<br>CACCC**C**T**T**GAGGCGCCTGAGCCTCGAAAGCCCGTGACAGCTCAGGAACGCCAG
**A27**	TCCGTGGACAT<br>GACCTGTGAggg	GCGACAGTGTATGGGTTCTTCAAGGGCCTAGCTCAGGAGCTCTCTTTCTTGCAGA**G**<br>GCACGAGTTCTC**T**GTGGACATGACCTG**C**GAGGGCTGTGCTGAAGCCGTCTCCAG
**A43**	GCAGAAGCAC<br>GAGTTCTCCGtg<br>g	GCGACAGTGTATGGGTTCTTCAAGGGCCTAGCTCAGGAGCTCTCTTTCTTGCAGA**G**<br>GCACGAGTTCTC**T**GTGGACATGACCTGTGAGGGCTGTGCTGAAGCCGTCTCCAG
**T77A**	CTGGGAGGACG<br>GGCTGACCGggg	GTCAGGAGCACAAGTACCTGCTCGGGAACGCCCC**A**GTCAGCCCG**G**CCTCCCAGA<br>AGCTCAAACGGAAGGCGGAGCAGGACCCCG
**T77D**	CTGGGAGGACG<br>GGCTGACCGggg	GTCAGGAGCACAAGTACCTGCTCGGGAACGCCCC**A**GTCAGCCCG**GA**CTCCCAGA<br>AGCTCAAACGGAAGGCGGAGCAGGACCCCG

Table S2. ***CRISPR component concentrations for each experimental condition.*** A donor in the same-sense-as the guide (S) was used for all but one of the single donor experiments. That exception was for *Magi1* guide M79 in which we used a donor complementary-to the guide (C), as well as one with the same-sense-as the guide (S). In the *Terf2ip* experiments, two donors were used at different ratios, both complementary-to the guide.

Project		[sgRNA]	[DNA1]	[DNA2]	DNA type	[Sp Cas9]	Cas9 type
Magi1-S733A	M79R	15 ng/µl	10 ng/µl (C)		ssODN	50 ng/µl	protein
Magi1-S733A	M79	10 ng/µl	10 ng/µl (S)		ssODN	50 ng/µl	protein
Magi1-S733A	M91	10 ng/µl	15 ng/µl (S)		ssODN	50 ng/µl	protein
Magi1-S733A	M79	5 ng/µl	15 ng/µl (S)		ssODN	10 ng/µl	mRNA
Magi1-S733A	M67	5 ng/µl	15 ng/µl (S)		ssODN	10 ng/µl	mRNA
Erk5-S496A	E84	5 ng/µl	15 ng/µl (S)		ssODN	50 ng/µl	protein
Erk5-S496A	E87	5 ng/µl	15 ng/µl (S)		ssODN	50 ng/µl	protein
Atox1-K3R	A27	10 ng/µl	15 ng/µl (S)		ssODN	50 ng/µl	protein
Atox1-K3R	A43	10 ng/µl	15 ng/µl (S)		ssODN	50 ng/µl	protein
Atox1-K3R	A27	10 ng/µl	10 ng/µl (S)		ssODN	50 ng/µl	protein
Terf2ip-S202A/D T77 (mixture 1, 3:1)		154 ng/µl	7 ng/µl (C)	2.6 ng/µl (C)	ssODN	50 ng/µl	protein
Terf2ip-S202A/D T77 (mixture 2, 9:1)		154 ng/µl	9 ng/µl (C)	1 ng/µl (C)	ssODN	50 ng/µl	protein

Table S3. ***Nested PCR primers used for sequencing.*** The primers described above were used for nested PCR of the genes noted in the first column. The primers noted as "First" were used for the initial PCR and the primers noted as "Second" were used in a subsequent nested PCR on the product produced by the first PCR reaction.

Gene		Primer sequence	Primer name
Magi1	First PCR forward primer	CCTCTATCTGACTACTTGACACC	mMagi1-7929F
	First PCR reverse primer	CCCTCTGTCTTTCTGCCAATC	mMagi1-8394R
	Second PCR forward primer	GTCGGTTTTCATACATGCTCC	mMagi1-8024F
	Second PCR reverse primer	GTTCTAAACTCATATGCACACGTG	mMagi1-8307R
MapK7	First PCR forward primer	TCTAGCAGGCTTCGGTCATTGTC	mMapk7-0295F
	First PCR reverse primer	TGCACCTGACACCGTTGATC	mMapk7-0734R
	Second PCR forward primer	TTCTCTCTCTTGTCGTCGCTTCTC	mMapk7-0388F
	Second PCR reverse primer	ACCTGACACCGTTGATCTGACTC	mMapk7-0728R
Atox1	First PCR forward primer	GTTGTATATGGTGGCATGGTGGTC	mAtox1-4565F
	First PCR reverse primer	TCTGTTGGGACTGCCTGTGATAC	mAtox1-5127R
	Second PCR forward primer	CCTCAAGCATCTGAACACGACTC	mAtox1-4835F
	Second PCR reverse primer	GACTAGGTTGGACTCACAGACACTTC	mAtox1-5064R
Terf2ip	First PCR forward primer	TGGATCGCAACGAGAAGCTG	mTerf2ip-1763F
	First PCR reverse primer	CGACACAGCGAAGAGACTCAAG	mTerf2ip-2382R
	Second PCR forward primer	AAGATGTGGCCATCCTGACCTAC	mTerf2ip-1913F
	Second PCR reverse primer	ACTTTCGCTTCGGACCTCAAC	mTerf2ip-2244R

Table S4. ***Estimation of guide RNA directed cutting efficiency.*** The percent DNA cut for each blastocyst was averaged in each experiment of the several experiments done for each guide/donor pair. The standard deviation (STD) was also calculated. Though the same guide was used for the *Terf2ip* experiments, two donors were simultaneously injected but at different ratios for each experiment. The average values between experiments are usually roughly similar for the same guide/donor pair while more obvious differences can be seen between different guide donor pairs.

Average percent DNA cut per blastocyst

Gene	Guide/ donor pair	Experiment	Average % ±STDV
Magi1	M67	exp #1 (n=3)	80±18
		exp #2 (n=2)	90±4
		exp #3 (n=14)	78±29
		total (n=19)	79±25
Magi1	M79	exp #1 (n=8)	85±19
		exp #2 (n=4)	70±47
		exp #3 (n=14)	79±26
		total=26	79±27
Magi1	M79R	exp #1 (n=4)	82±17
		exp #2 (n=15)	95±7
		total=19	93±11
Magi1	M91	exp #1 (n=3)	97±0
		exp #2 (n=14)	80±31
		total=17	83±29

Gene	Guide/ donor pair	Experiment	Average % ±STDV
Mapk7	E84	exp #1 (n=7)	53±35
		exp #2 (n=10)	20±32
		exp #3 (n=11)	31±24
		total (n=28)	32±32
	E87	exp #1 (n=12)	88±14
		exp #2 (n=7)	99±8
		total (n=19)	92±13
Atox1	A27	exp #1 (n=8)	26±43
		exp #2 (n=8)	91±17
		total (n=16)	58±46
	A43	exp #1 (n=8)	1±1
		exp #2 (n=6)	0±1
		total (n=14)	1±1
Terf2ip	3:1	exp 3:1 (n=9)	91±14
	9:1	exp 9:1 (n=13)	93±7

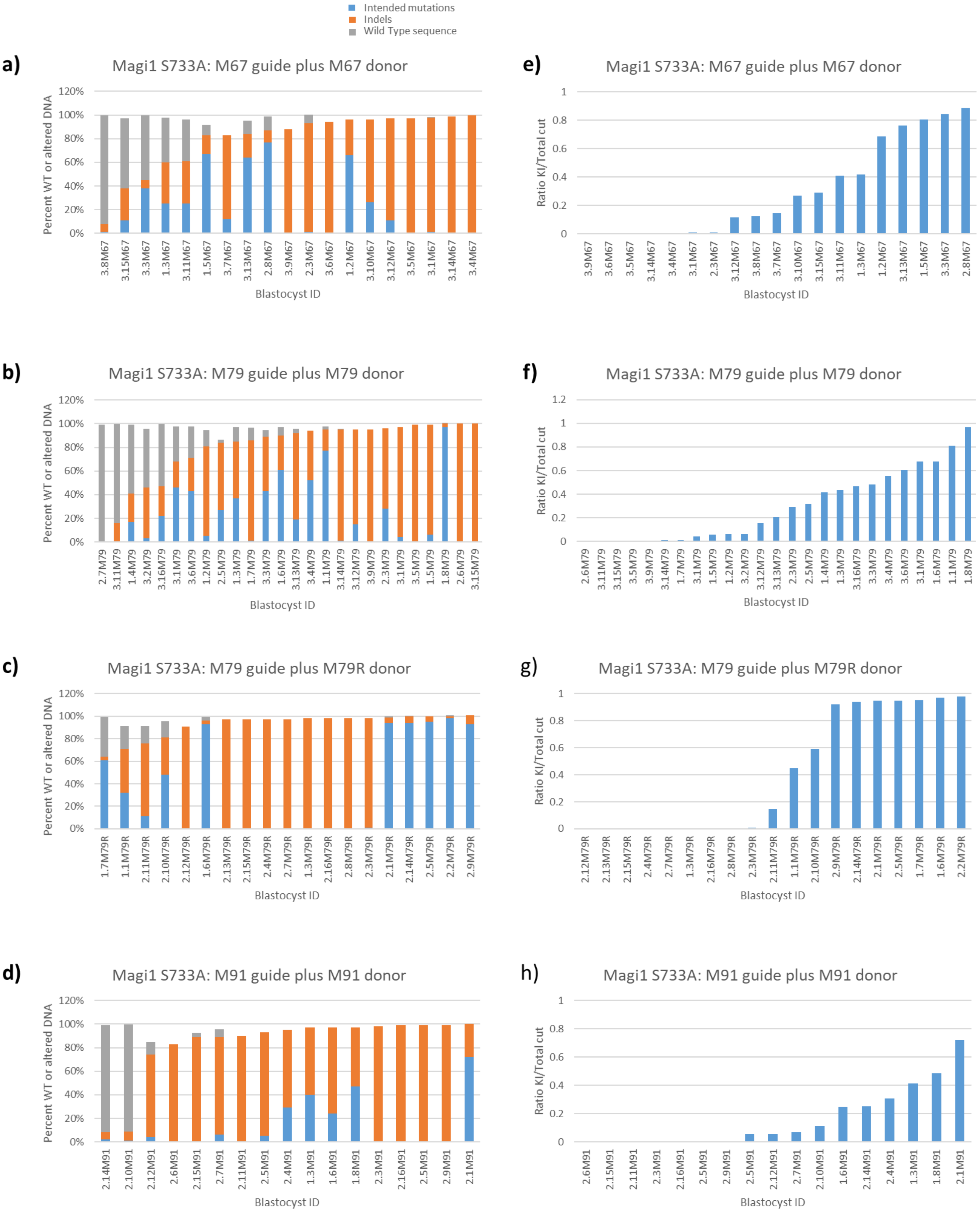

Figure S1. ***Pattern of guide RNA cutting compared to pattern of donor DNA integration for different guide/donor pairs in Magi1***. Bar graph of data from experiments with *Magi1* guide RNA M79 (a, b, e, f), M67 (c, g), and M91 (d, h) treated blastocysts. For each guide/donor pair, data from two or three separate experiments were combined. Each bar in a-d shows the percentage of intended mutations (blue), indel (orange), and WT (gray) for a single blastocyst. Each bar in e-h shows the ratio of desired

point mutation KIs per total cut genomic DNA for a single blastocyst. Bars are ordered from those with the lowest ratio of desired KI mutation per total cut to those with the highest ratio.

a) and e) Blastocysts treated with guide RNA M79 and same-sense donor DNA. Desired mutation is 18 bp from cut.

b) and f) Blastocysts treated with guide RNA M79 and complementary donor DNA. As with the same-sense, the desired mutation is 18 bp from cut.

c) and g) Blastocysts treated with guide RNA M67 and same-sense donor DNA. Desired mutation is 31 bp from cut.

d) and h) Blastocysts treated with guide RNA M91 and same-sense donor DNA. Desired mutation is 5 bp from cut.

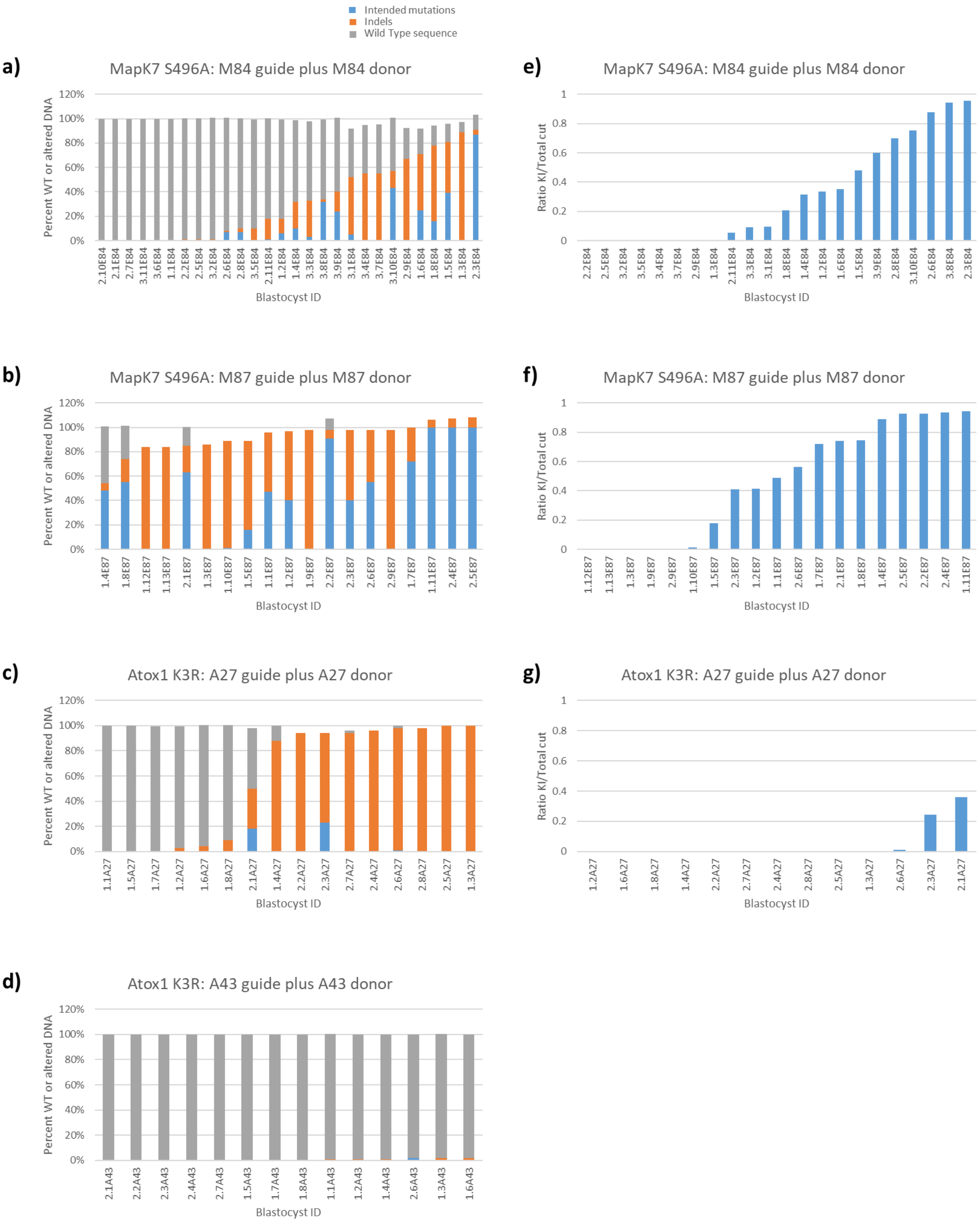

Figure S2. *Different patterns of cutting, similar pattern of integration from different guide/donor pairs in different genes.* Bar graph of data from experiments with *MapK7* guide RNA M79 (a, e) and M67 (b, f), and *Atox1* guide RNA M79 (c, g) and M67 (d) treated blastocysts. For each guide/donor pair, data from two or three separate experiments were combined. Each bar in a-d shows the percentage of intended mutations (blue), indel (orange), and WT (gray) for a single blastocyst. Each bar in e-g shows the ratio of desired point mutation KIs per total cut genomic DNA for a single blastocyst. Bars are

ordered from those with the lowest ratio of desired KI mutation per total cut to those with the highest ratio. The total cut for each blastocyst in the *Atox1* M67 experiments ranged from 0 to 2%. As these percents are within the assay error rate, the ratios of desired KIs per total cut were not calculated for *Atox1* M67.

a) and e) Blastocysts treated with guide RNA E84 and same-sense donor DNA. Desired mutation is 10 bp from cut.

b) and f) Blastocysts treated with guide RNA E87 and same-sense donor DNA. Desired mutation is 8 bp from cut.

c) and g) Blastocysts treated with guide RNA A27 and same-sense donor DNA. Desired mutation is 27 bp from cut.

d) Blastocysts treated with guide RNA A43 and same-sense donor DNA. Desired mutation is 11 bp from cut.
